# Coupling plankton and cholera dynamics: Insights into outbreak prediction and practical disease management

**DOI:** 10.1371/journal.pcbi.1013523

**Published:** 2025-09-29

**Authors:** Biplab Maity, Swarnendu Banerjee, Abhishek Senapati, Jon Pitchford, Joydev Chattopadhyay

**Affiliations:** 1 Agricultural and Ecological Research Unit, Indian Statistical Institute, Kolkata, India; 2 Dutch Institute for Emergent Phenomena, Institute for Biodiversity and Ecosystem Dynamics, Korteweg-de Vries Institute for Mathematics, University of Amsterdam, Amsterdam, The Netherlands; 3 Copernicus Institute of Sustainable Development, Utrecht University, Utrecht, The Netherlands; 4 Saw Swee Hock School of Public Health, National University of Singapore, Singapore; 5 Department of Biology, University of York, Wentworth Way, York, United Kingdom; 6 Department of Mathematics, University of York, Heslington, York, United Kingdom; Fundação Getúlio Vargas: Fundacao Getulio Vargas, BRAZIL

## Abstract

Despite extensive control efforts over the centuries, cholera remains a globally significant health issue. Seasonal emergence of cholera cases has been reported, particularly in the Bengal delta region, which is often synchronized with plankton blooms. This phenomenon has been widely attributed to the commensal interaction between *Vibrio cholerae* and zooplankton in aquatic environments. The role of plankton dynamics in cholera epidemiology has been acknowledged but remains poorly understood, and consequently, its importance in effective policymaking is largely overlooked. To this end, we propose and analyze a novel compartment-based transmission model that integrates phytoplankton-zooplankton interactions into a human-bacteria cholera framework. Our study shows that, beyond the reproduction number, the relative contribution of bacterial versus zooplankton-mediated transmission plays a crucial role in shaping epidemic progression and severity. In presence of zooplankton-mediated transmission, an outbreak with a delayed and lower peak may still result in a larger overall outbreak size. Additionally, contrary to common intuition, even for a large and early outbreak, the epidemic overshoot may intensify due to the maintenance of lower-level infections during the post-peak phase. Furthermore, our analysis reveals that the timing of filtration-like interventions can be strategically guided by ecological indicators, such as phytoplankton blooms. Our study underscores the importance of incorporating ecological aspects in epidemiological research to better predict and manage disease outbreaks.

## Author summary

In the Bengal Delta region, cholera outbreaks re-emerge seasonally, often synchronized with plankton blooms. Although it is known that the disease is caused by *Vibrio cholerae*, a bacterium associated with zooplankton, the role of plankton ecology in cholera spread is not yet well understood. To address this, we have developed a novel disease transmission model that combines plankton interactions with the traditional human-bacteria cholera framework. Our findings show that when cholera spreads through zooplankton, infections can persist at a low level for a long time, leading to a large overall outbreak size, even when the number of cases at peak is not very high. The results also indicate the possibility that a large portion of the susceptible population is infected beyond the peak of infection. This underlines the need to maintain control measures during the post-peak period in regions with evidence of *Vibrio cholerae* reservoirs. We also show that not only the basic reproduction number, but also the strength of transmission pathways, plays a key role in determining the severity of an outbreak. We have also found that ecological indicators, such as phytoplankton blooms, can help to guide the timing of disease control measures like water filtration.

## 1. Introduction

Cholera, while treatable, remains a major global health emergency, with an estimated 4 million annual cases including 1,43,000 deaths globally [1,2]. Despite uncertainties in case reporting, recent methodologies indicate between 470,000-790,00 cholera cases, and up to 5,000 deaths, annually in the early 2020s [3,4]. The majority of these cases are in Africa, but the presence of cholera in Afghanistan, Yemen, Pakistan, Haiti, Bangladesh, and India underlines its importance in emerging and developing countries [5–9]. Country-specific factors and a critical shortage of oral vaccines in 2023 have led the WHO to categorize the resurgence of cholera as a grade 3 emergency [4].

The consistent seasonal re-emergence of cholera in the Bengal Delta region over the past decades has been attributed to seasonal plankton blooms [10–14]. It has been widely established that *Vibrio cholerae* (*V. cholerae*), the causative bacteria, is associated with plankton [15–18]. The detection of *V. cholerae* associated with zooplankton cells along the coasts of Brazil and Mexico suggests that the ecological relationship between bacteria and plankton is widespread [19,20]. Copepods, a crustacean zooplankton, are the largest known natural reservoir of the *V. cholerae* and have been implicated as a potential vector [15,21]. Experimental studies by Turner et al. [22] and Rawlings et al. [23] in natural estuarine and coastal ecosystems of Georgia, USA, as well as in the Bengal delta region found a significant correlation between *V. cholerae* density and copepod abundance. Studies indicate that a single copepod can carry 10^4^ to 10^6^ bacterial cells [12,24–26].

*Vibrios* colonize the gut of the zooplankton and the surface biofilms, where they can multiply rapidly under favorable nutrient conditions [12,18,27–29]. Further, the zooplankton protects the *Vibrios* from grazing as well as from chemical disinfectants, significantly prolonging bacterial survival compared to free-living cells [12,30–33]. This relationship between the zooplankton and the *Vibrios*, where only the former benefits from the association, is known as commensalism [18]. The empirical study by [34] asserted that, at high concentrations, *Vibrios* are more likely to attach to zooplankton cells rather than remain free in the surrounding water. As a result, exposure to commensal zooplankton could potentially lead to the consumption of a large bacterial inoculum. This underscores the fundamental role of zooplankton in cholera dynamics and emphasizes the importance of studying plankton ecology, particularly in regions with evidence of *V. cholerae* reservoirs [35].

Mathematical models have been proven to be an important and reliable tool for understanding cholera transmission mechanisms and guiding policymaking during several outbreaks [36–41]. In spite of numerous empirical studies over the past decades linking plankton abundance to increased cholera infections [10–14,29,34,42], a mechanistic model that explicitly connects the ecology of plankton to *V. cholerae* transmission is still lacking. A recent study by Kolaye et al. [43] considered commensalism between bacteria and phytoplankton, focusing on bacterial metabolism changes. However, this study neither included any explicit compartment for commensal plankton nor studied transmission via plankton. To address this gap, we have developed a novel compartmental transmission model that integrates the phytoplankton-zooplankton interactions with the classical susceptible-infected-recovered-bacteria (SIRB) cholera model [44]. Our model includes a separate compartment for *Vibrio*-associated zooplankton cells and accounts for two transmission routes: one for free-living bacterial cells and another for bacteria-associated zooplankton. The role of zooplankton-mediated transmission in short-term epidemic outbreaks has been investigated. We assess the relative importance of transmission routes in shaping epidemic progression. Additionally, we have explored the epidemic overshoot phenomenon, which is linked to the post-peak severity of outbreaks. Further, we evaluate the effect of filtration as a practical disease management measure.

## 2. Methods

### 2.1. Model formulation

**Fig 1:**
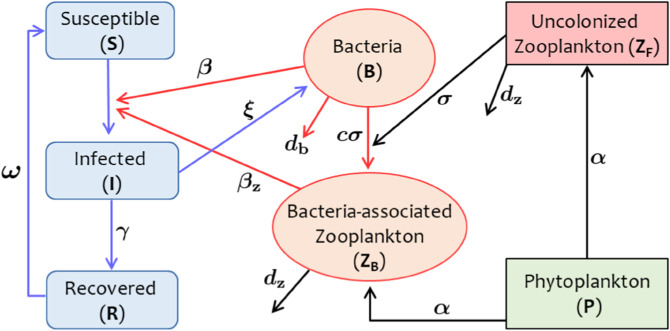
Coupled plankton-cholera model structure. Model schematic illustrating the linkage between phytoplankton-zooplankton interactions and the classical susceptible-infected-recovered-bacteria (SIRB) cholera model through the commensal association between *Vibrio cholerae* and zooplankton. Model parameters and their corresponding values are given in Table 1.

Our approach combines established SIR models for disease transmission with well-developed models for plankton dynamics, using a minimal set of biologically justifiable and quantifiable assumptions. Fig 1 and Table 1 summaries this approach. This framework facilitates a range of analyses, including the characterization of steady states and transient dynamics, computational sensitivity analysis, and the evaluation of control scenarios, across a range of ecologically relevant time scales.

**Table 1:** Description of parameters for the model (2.1).

Parameters	Description	Value	Reference
Λ	Constant recruitment rate of human population	$6.85\;\times \;10^{-5}\;\times \;N_{0}$ person ${\text{day}}^{-1}$	[49]
*N*_0_	Initial population size	220,000	[49]
*β*	Transmission rate via bacteria	0.214 ${\text{day}}^{-1}$	[38,47,50]
$\beta_{z}$	Transmission rate via zooplankton	variable but less than *β*	Investigated (Sect A in S1 Text)
*h*_*b*_	Half-saturation constant of bacterial transmission	10^9^ cells ${\text{L}}^{-1}$	[47]
*h*_*z*_	Half-saturation constant of transmission via zooplankton	20 (mg dw) ${\text{L}}^{-1}$	Investigated (Sect A in S1 Text)
*μ*	Natural death rate of human	$3.8\times 10^{-5}$ ${\text{day}}^{-1}$	[51,52]
*ω*	Rate of immunity loss of recovered individuals	0.00092 ${\text{day}}^{-1}$	[49,53]
*γ*	Recovery rate of infected human	$0.2\;{\text{day}}^{-1}$	[38,49]
*δ*	Disease induced mortality rate of human	0.013 ${\text{day}}^{-1}$	[36,43,50]
ξ	Bacteria shedding rate of infected humans	10-10^4^ cells ${\text{L}}^{-1}$ ${\text{day}}^{-1}$ per person	[44,50,54]
*d*_*b*_	Removal rate of the bacteria (birth - death)	0.33 ${\text{day}}^{-1}$	[43,44]
*σ*	Rate of bacteria-zooplankton association	0.005-0.1 ${\text{day}}^{-1}$	Investigated (Sect 3.1)
*c*	Colonization coefficient of bacteria	$5\times 10^{7}$ ${\text{cells (mg dw)}}^{-1}$	Investigated (Sect A in S1 Text)
*h*_*m*_	Half saturation constant for bacteria-zooplankton association	10^7^ cells ${\text{L}}^{-1}$	Sect A in S1 Text
*η*	Conversion coefficient	0.6	[55]
*α*	Predation rate	0.4 day−1	[55]
*h*_*p*_	Half saturation constant of phytoplankton	0.6 (mg dw) ${\text{L}}^{-1}$	[55]
*d*_*z*_	Death rate of zooplankton	0.06 ${\text{day}}^{-1}$	[56–58]
*r*_*p*_	Growth rate of phytoplankton	0.5 ${\text{day}}^{-1}$	[55]
*K*	Carrying capacity of phytoplankton	0.95 (mg dw) ${\text{L}}^{-1}$	[59]

The total human population at time *t*, *N*(*t*), is divided into three compartments: susceptible (*S*(*t*)), infected (*I*(*t*)) and recovered (*R*(*t*)). Earlier models have typically included a single compartment for the bacterial population in the water column [44,45]. However, the commensal relationship between *V. cholerae* and zooplankton results in the bacteria being associated with zooplankton cells, which can facilitate human infection upon exposure [15,33]. This phenomenon motivates the inclusion of an explicit compartment for bacteria-associated zooplankton (*Z*_*B*_(*t*)), which is formed when free-living bacteria (*B*(*t*)) attaches to uncolonized zooplankton (*Z*_*F*_(*t*)). Hence, $Z(t)=Z_{F}(t)\;+\;Z_{B}(t)$ is the total zooplankton density at time *t*. The force of *Vibrio*-zooplankton commensalism is represented by the term $c\sigma \frac{B}{h_{m}\;+\;B}$. Here, *σ* denotes the rate of bacteria-zooplankton association and *h*_*m*_ is the half-saturation constant of the association. The parameter *c* represents the average number of *Vibrio* cells per zooplankton, which we term the ‘colonization coefficient’. Since phytoplankton (*P*(*t*)) governs zooplankton (*Z*(*t*)) abundance, we consider the dynamics of phytoplankton-zooplankton using a well-studied Rosenzweig–MacArthur type model [46].

Susceptible humans become infected through exposure to free-living bacteria (via water contamination), *B*(*t*), with a force of infection $\lambda_{B}=\frac{\beta B}{h_{b}\;+\;B}$ and *Vibrio*-associated zooplankton, *Z*_*B*_(*t*), with a force of infection $\lambda_{Z}=\frac{\beta_{z}Z_{B}}{h_{z}\;+\;Z_{B}}$. Here, *h*_*b*_ and *h*_*z*_ represent the half-saturation constant for transmission via bacterial and zooplankton routes, respectively. Note that *h*_*z*_ depends on the pathogen load of commensal zooplankton and is inversely proportional to the bacterial colonization coefficient, *c*. The use of Holling type-II transmission terms follows existing literature [44,45,47]. Our model, which includes transmission via both free-living bacteria and bacteria-associated zooplankton, is given as follows (see Fig 1 for schematic).

$$\frac{dS}{dt}=\Lambda -\frac{\beta SB}{h_{b}+B}-\frac{\beta_{z}SZ_{B}}{h_{z}+Z_{B}}-\mu S+\omega R,\;\frac{dI}{dt}=\frac{\beta SB}{h_{b}+B}+\frac{\beta_{z}SZ_{B}}{h_{z}+Z_{B}}-(\gamma +\mu +\delta )I,\;\frac{dR}{dt}=\gamma I-(\mu +\omega )R,\;\frac{dB}{dt}=\xi I-d_{b}B-c\sigma \frac{BZ_{F}}{h_{m}+B},\;\frac{dZ_{B}}{dt}=\sigma \frac{BZ_{F}}{h_{m}+B}-d_{z}Z_{B},\;\frac{dZ_{F}}{dt}=\eta \frac{\alpha P(Z_{F}+Z_{B})}{h_{p}+P}-d_{z}Z_{F}-\sigma \frac{BZ_{F}}{h_{m}+B},\;\frac{dP}{dt}=r_{p}P(1-\frac{P}{K})-\frac{\alpha P(Z_{F}+Z_{B})}{h_{p}+P}.$$ (2.1)

Infected individuals either recover at a rate *γ* or die due to infection at a rate *δ* and contribute bacterial cells into the environment through excretion at a rate ξ over their infectious period. As cholera does not confer life-long immunity [45,48], recovered individuals, although initially immune to the pathogen, eventually lose immunity at a rate *ω* and replenish into the susceptible compartment. Λ denotes the recruitment rate of susceptible individuals through birth and immigration, while each class experiences a natural death rate *μ*. Here, *d*_*b*_ represents the rate of loss of free-living bacteria, accounting for both mortality and the decline in vitality. Phytoplankton grows at a rate *r*_*p*_ up to its maximum achievable density, known as the carrying capacity, *K*. The maximal grazing rate of the zooplankton on phytoplankton is *α* with a conversion coefficient *η*. Here, *d*_*z*_ and *h*_*p*_ refer to the zooplankton death rate and the half-saturation concentration of zooplankton grazing, respectively.

Note that the plankton dynamics, represented by the last three equations in model (2.1), is independent of the human-bacteria (SIRB) dynamics (see Sect C in S1 Text). This independence implies that the bacteria-zooplankton association does not influence overall plankton density, which is representative of the commensal interaction. However, this association leads to the formation of *Z*_*B*_ and reduces free-living bacterial density, thereby affecting human-bacteria dynamics. Note that we do not consider direct human-to-human transmission here, as it has been shown that the aquatic environment plays a decisive role in the survival and transmission of pathogens during cholera outbreaks in several regions [15]. If the bacteria-zooplankton association is excluded (i.e. σ=0), our model (2.1) resembles previous SIRB cholera models, such as those used in [44,48].

## 3. Results

Our results are presented according to the ecologically and management-motivated time scales relevant to this study. The main body of work (Sect 3.1) analyses cholera outbreaks in human populations over a short time scale, where human population and immunity dynamics can be neglected (i.e. μ,Λ=0 and ω=0 in model (2.1)). We then contextualize these findings in the context of practical interventions connected to water filtration (Sect 3.2).

### 3.1. Outbreak dynamics

To remove the effect of transient plankton dynamics on disease outbreak, we consider the coexistent steady-state plankton densities as the initial condition for phytoplankton ($P_{0}=P^{*}$) and zooplankton ($Z_{F_{0}}=Z^{*},Z_{B}=0$) in model (2.1) (see Sect C in S1 Text for P^*^,Z^*^). This approach is reasonable in light of the following: (i) the transient plankton dynamics has no biological significance in the context of an outbreak, and (ii) the outbreak does not alter the plankton dynamics. Note that the zooplankton-free equilibrium ($Z_{F}=Z_{B}=0$) of the phytoplankton-zooplankton system is not relevant in the context of our study.

The necessary condition for the initial growth of an outbreak in the presence of both transmission routes is given by (see Sect D in S1 Text)

$${ℛ}_{0_{\text{BZ}}}^{\text{out}}={ℛ}_{0_{B}}^{\text{out}}+{ℛ}_{0_{Z}}^{\text{out}}\;=\frac{\xi N_{0}}{\left(\gamma +\delta )(d_{b}+c\sigma \frac{Z^{*}}{h_{m}}\right)}[\frac{\beta }{h_{b}}+\frac{\beta_{z}}{h_{z}}\sigma \frac{Z^{*}}{d_{z}h_{m}}]>1$$ (3.1)

where


$$\begin{array}{cc} {ℛ}_{0_{B}}^{\text{out}} & =\frac{\xi N_{0}}{\left(\gamma +\delta )(d_{b}+c\sigma \frac{Z^{*}}{h_{m}}\right)}\frac{\beta }{h_{b}},\\ {ℛ}_{0_{Z}}^{\text{out}} & =\frac{\xi N_{0}}{\left(\gamma +\delta )(d_{b}+c\sigma \frac{Z^{*}}{h_{m}}\right)}\frac{\beta_{z}}{h_{z}}\sigma \frac{Z^{*}}{d_{z}h_{m}}. \end{array}$$

Here, ${ℛ}_{0_{\text{BZ}}}^{\text{out}}$ denotes the basic reproduction number (see Sect E in S1 Text for the basic reproduction number in the long-term scenario). Also, ${ℛ}_{0_{B}}^{\text{out}}$ and ${ℛ}_{0_{Z}}^{\text{out}}$ represent contributions associated with the free-living bacterial route and the bacteria-associated zooplankton route, respectively, to the initial outbreak growth.

#### 3.1.1. Impact of bacteria-zooplankton association on ${ℛ}_{0_{\text{BZ}}}^{\text{out}}$.

In the absence of the bacteria-zooplankton (*B*-*Z*) association (i.e. σ=0), the condition for the initial outbreak growth ${ℛ}_{0}^{\text{out}}={ℛ}_{0_{\text{BZ}}}^{\text{out}}{|}_{\sigma =0}=\frac{\xi N_{0}}{d_{b}(\gamma \;+\;\delta )}\frac{\beta }{h_{b}}>1$, aligns with the classic SIRB cholera model [44]. The colonization of zooplankton by bacterial cells reduces the free-living bacterial density, thereby lowering ${ℛ}_{0_{B}}^{\text{out}}$. This association simultaneously increases ${ℛ}_{0_{Z}}^{\text{out}}$ through the transmission via commensal zooplankton. As a result, for a specific *β*, the *B*-*Z* association can lead to an increase or decrease of ${ℛ}_{0_{\text{BZ}}}^{\text{out}}$ relative to ${ℛ}_{0}^{\text{out}}$ depending on *σ* and $\beta_{z}$.

**Fig 2:**
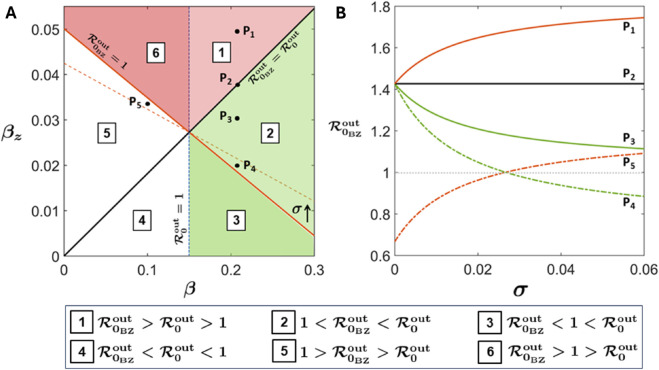
Impact of bacteria-zooplankton association on ${ℛ}_{0_{\text{BZ}}}^{\text{out}}$. (A) Effect of the transmission rates *β*, $\beta_{z}$. The red and black lines depict ${ℛ}_{0_{\text{BZ}}}^{\text{out}}=1$ and ${ℛ}_{0_{\text{BZ}}}^{\text{out}}={ℛ}_{0}^{\text{out}}$, respectively. The vertical dashed line indicates ${ℛ}_{0}^{\text{out}}=1$, corresponding to the absence of the *B*-*Z* association. An increase in *σ* expands regions 6 and 3 at the expense of 5 and 2, respectively, as indicated by the red-dashed line. (B) The effect of the association rate (*σ*) on points *P*_1_-*P*_5_, which belong to different regions demonstrated in (A).

The two-parameter space (*β*-$\beta_{z}$) can be divided into six regions using the three lines, ${ℛ}_{0}^{\text{out}}=1$ (dashed vertical line), ${ℛ}_{0_{\text{BZ}}}^{\text{out}}=1$ (red line), and ${ℛ}_{0_{\text{BZ}}}^{\text{out}}={ℛ}_{0}^{\text{out}}$ (black line) (Fig 2A). While, in region 1 and 6, the *B*-*Z* association increases ${ℛ}_{0_{\text{BZ}}}^{\text{out}}$ compared to ${ℛ}_{0}^{\text{out}}$, in 2 and 3, it decreases the same. Here, the decrease in ${ℛ}_{0_{\text{BZ}}}^{\text{out}}$ can be achieved with reduced $\beta_{z}$, which signifies the reduced exposure to zooplankton contaminated water. It is interesting to note that in 6, an outbreak cannot initiate without the *B*-*Z* association, highlighting the significance of zooplankton-driven route. This contrasts with the behavior in 3, where an outbreak that can grow without this association decays in its presence. In regions 4 and 5, the outbreak fails to grow irrespective of the presence or absence of the *B*-*Z* association, making it insignificant in the context of our study. For a lower *β*, a relatively high $\beta_{z}$ can still trigger an outbreak when ${ℛ}_{0_{\text{BZ}}}^{\text{out}}>1$. This implies that zooplankton-mediated transmission can compensate for a low transmission rate via free-living bacteria.

For any point in 1, e.g. *P*_1_, an increased bacteria-zooplankton association rate (*σ*) always increases ${ℛ}_{0_{\text{BZ}}}^{\text{out}}$ (red line Fig 2B). Further, slope of the line ${ℛ}_{0_{\text{BZ}}}^{\text{out}}=1$ decreases with increasing *σ* (red-dashed line in Fig 2A) (see Sect D in S1 Text for details). As a result, regions 3 and 6 expand at the expense of 2 and 5, respectively. Consequence of this can be observed by following the fate of the outbreak on increasing *σ* at points *P*_4_ and *P*_5_. At *P*_4_, where the outbreak initially grows (${ℛ}_{0_{\text{BZ}}}^{\text{out}}>1$), the system is driven to the region where the outbreak decays (${ℛ}_{0_{\text{BZ}}}^{\text{out}}<1$) with increasing *σ* (green-dashed line in Fig 2B). The converse is true for the point *P*_5_ as shown by red-dashed line in Fig 2B. Additionally, there are some points in 2, e.g. *P*_3_, for which increasing *σ* will decrease ${ℛ}_{0_{\text{BZ}}}^{\text{out}}$ while still maintaining it above 1 (green line in Fig 2B). The black (*P*_2_) line in Fig 2B represents the scenario ${ℛ}_{0_{\text{BZ}}}^{\text{out}}={ℛ}_{0}^{\text{out}}$ under any association rate (*σ*). Moreover, from Fig 2B, it is important to note that for a fixed *β*, changes in $\beta_{z}$ (e.g., *P*_1_-*P*_4_) lead to relatively larger variations in ${ℛ}_{0_{\text{BZ}}}^{\text{out}}$ under a high *σ* compared to a lower one. This can be explained by the fact that under favorable conditions, large number of *Vibrio*-associated zooplankton cells significantly impact the disease spread. We use global sensitivity analysis to show that the above result holds true for all values of *β* (see Sect D in S1 Text).

Notably, in this study, we always consider $d_{z}<d_{b}$, which is well supported by ecological evidences [43,44,56–58]. An increase in *d*_*z*_ increases the slope of the ${ℛ}_{0_{\text{BZ}}}^{\text{out}}={ℛ}_{0}^{\text{out}}$ line, thereby reducing the region 1 (see S1 Fig). This indicates a diminished effect of transmission via zooplankton as the persistence of bacterial cells in association with zooplankton is reduced.

#### 3.1.2. Relative contribution of transmission routes.

The ratio of contributions to the basic reproduction number (${ℛ}_{0_{\text{BZ}}}^{\text{ out}}$) from zooplankton (*Z*_*B*_) and bacterial (*B*) routes can be expressed as ${ℛ}_{0_{Z}}^{\text{out}}/{ℛ}_{0_{B}}^{\text{ out}}=(\sigma Z^{*}h_{b}\beta_{z})/(d_{Z}h_{z}h_{m}\beta )$. Environmental factors, such as fluctuations in the temperature of the coastal sea surface, salinity, pH, heatwave, rainfall, floods, and ecological events such as plankton blooms, can influence the bacteria-zooplankton association [28,30,60], causing one transmission route to become more prominent over the other. Therefore, it is crucial to investigate for a fixed ${ℛ}_{0_{\text{BZ}}}^{\text{out}}$, how altering relative contributions (${ℛ}_{0_{B}}^{\text{out}}$, ${ℛ}_{0_{Z}}^{\text{out}}$) through different transmission routes can impact disease progression and overall disease burden. To this aim, we keep ${ℛ}_{0_{\text{BZ}}}^{\text{out}}={ℛ}_{0}^{\text{out}}>1$, which always implies $\beta_{z}=\frac{ch_{z}d_{z}}{h_{b}d_{b}}\beta$ (black line in Fig 2A). For a fixed *β*, corresponding to each pair of relative contributions (${ℛ}_{0_{B}}^{\text{out}}$, ${ℛ}_{0_{Z}}^{\text{ out}}$), the unique association rate *σ* is given by (see Sect D in S1 Text for details)


$$\sigma =\frac{d_{b}h_{m}}{cZ^{*}}(\frac{{ℛ}_{0_{\text{BZ}}}^{\text{out}}}{{ℛ}_{0_{B}}^{\text{out}}}-1)=\frac{d_{b}h_{m}}{cZ^{*}}\frac{{ℛ}_{0_{Z}}^{\text{out}}}{{ℛ}_{0_{B}}^{\text{out}}}.$$

**Fig 3:**
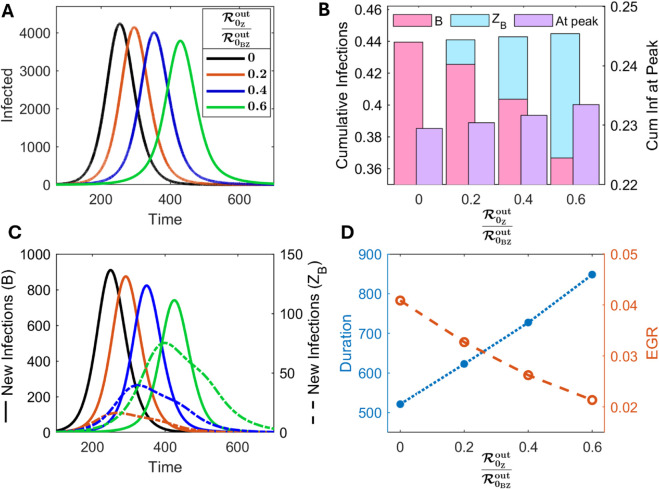
Effect of changing the relative contribution of transmission routes while keeping ${ℛ}_{0_{\text{BZ}}}^{\text{out}}$ fixed. (A) Active infections, (B) cumulative infections (proportion of the population), (C) new infections via the *B* (solid lines) and *Z*_*B*_ (dashed lines) route, and (D) epidemic duration (blue dotted) and initial epidemic growth rate (EGR) (red dashed). Increasing ${ℛ}_{0_{Z}}^{\text{out}}$ decreases initial EGR, delays and lowers the peak, but increases cumulative infections both at the peak and at the end of the outbreak.

Now, we compare outbreak trajectories by varying *σ* that captures scenarios for different relative contributions from the both routes. We examine various characteristics of these trajectories such as peak values, peak timing, cumulative infections, and epidemic duration (see Fig 3).

In the absence of *Vibrio*-zooplankton association (σ=0), transmission only via the bacterial route drives the prevalence with a relatively higher and earlier peak, followed by a steeper decline (black line in Fig 3A). When the *Z*_*B*_ route contributes a moderate to high proportion (20% to 60%), the outbreak progresses over a longer duration with delayed and reduced epidemic peak (Fig 3A and 3D). However, it results in a slightly increased proportion of cumulative infections both at peak and at the end of the outbreak, compared to the σ=0 scenario (Fig 3B). The delayed dynamics in the presence of the *B*-*Z* association arise because the contribution from the *Z*_*B*_ route leads to a comparatively slower initial epidemic growth rate (EGR) (Fig 3D and Sect D in S1 Text for EGR calculations). The zooplankton-mediated transmission route can be viewed as a delayed transmission pathway, as the zooplankton have to first be colonized by bacteria cells before transmitting the infection to susceptible persons. The increase in cumulative infections at the peak in the presence of the zooplankton route means greater depletion of susceptible population before the peak and indicates an increased threshold of herd immunity for the same ${ℛ}_{0_{\text{BZ}}}^{\text{out}}$ (Fig 3B). These observations emphasize that predictions about the outbreak trajectories and disease severity can not be precisely determined only by analyzing ${ℛ}_{0_{\text{BZ}}}^{\text{out}}$, the contribution of transmission routes is also important.

In spite of the increasing cumulative infections, the *Z*_*B*_ route produces fewer new infections compared to the *B* route in each case (Fig 3B and 3C). In fact, the epidemic peak’s size and timing are primarily determined by the *B* route, which is responsible for the majority of new infections (Fig 3C). Increasing ${ℛ}_{0_{Z}}^{\text{out}}$ leads to reduced density of *B* cells which results in a lower peak of infections. This occurs because, when ${ℛ}_{0_{\text{BZ}}}^{\text{out}}={ℛ}_{0}^{\text{out}}$, (i.e. $\beta_{z}=\frac{ch_{z}d_{z}}{h_{b}d_{b}}\beta$, black line in Fig 2A), we always have $\beta_{z}<\beta$ as $d_{z}<d_{b}$ and thus, the *Z*_*B*_ route always provides a lower force of infection than the *B* route.

#### 3.1.3. Nature of outbreak trajectories.

**Fig 4:**
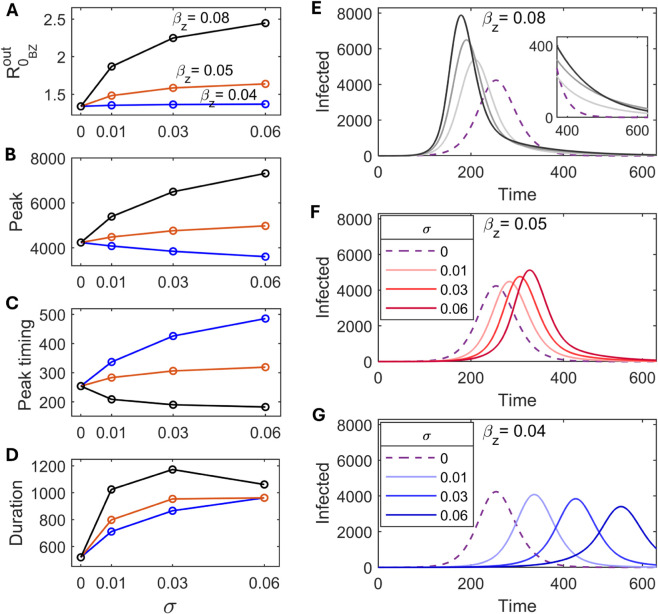
Influence of association rate and zooplankton-mediated transmission on epidemic dynamics. ${ℛ}_{0_{\text{BZ}}}^{\text{out}}$ (A), peak infections (B), peak timing (C), epidemic duration (D) and the outbreak trajectories (E-G) under varying bacteria-zooplankton association rate (σ) for different transmission rates via zooplankton ($\beta_{z}$) within region 1 of Fig 2A.

In this section, we investigate the nature of the outbreak trajectories within region 1 of Fig 2A while varying the bacteria-zooplankton association rate (*σ*) for different zooplankton-mediated transmission rate ($\beta_{z}$). For high $\beta_{z}=0.08$, an increased *σ* leads to both earlier (EGR increases) and higher peaks compared to σ=0 case (Fig 4B, 4C, 4E and S2 Fig). When $\beta_{z}$ is low ($\beta_{z}=0.04$), a delayed and lower peak is observed. However, for intermediate $\beta_{z}$ (=0.05), while the peak is delayed, the number of infections at peak increases (Fig 4B, 4C, 4F and 4G). The decrease in EGR with increasing *σ* for both $\beta_{z}=0.04$ and $\beta_{z}=0.05$ explains the delayed dynamics in these scenarios (see S2 Fig). Since an outbreak with a delayed and lower peak can still result in a larger outbreak size (due to higher ${ℛ}_{0_{\text{BZ}}}^{\text{out}}$ at increased *σ*, Fig 4A), it may not be easy to predict the outbreak size based on peak value and peak timing when zooplankton-mediated transmission is involved (Fig 4G). For all of the above scenarios, the epidemic duration increases noticeably, compared to the σ=0 case (Fig 4D).

Under fixed σ=0.03, an increased $\beta_{z}$ leads to an increased duration of epidemic in spite of earlier and larger peak value (Fig 4 and S3 Fig). This is due to the fact that increasing $\beta_{z}$ results in a considerably slower asymptotic convergence of the infection trajectory to the disease-free state after the peak infections, thus prolonging the duration of the outbreak (for example, see Fig 4E). This is in contrary to the usual notion whereby an epidemic trajectory with a higher reproduction number should results in a relatively larger and faster peak followed by a quicker decline. This observation underscores the importance of zooplankton in promoting pathogen persistence during inter-epidemic periods by serving as a *V. cholerae* reservoir. The maintenance of lower-level infections for a long period continues to influence the number of infections after the peak or equivalently after achieving the herd immunity threshold. In this context, the next subsection explores the epidemic overshoot phenomenon, which accounts for outbreak severity in the post-peak phase.

#### 3.1.4. Epidemic overshoot.

Epidemic overshoot refers to the proportion of the population that becomes infected after the peak of infection has passed, i.e. during the post-peak phase. It is equivalent to the difference between the herd immunity threshold and the attack rate (i.e. proportion of the infected population) [61]. Notably, the ratio of overshoot to attack rate ($\rho_{\text{OA}}$) is an important metric for quantifying the fraction of total infections occurring at the overshoot phase [62]. In the absence of bacteria-zooplankton association (σ=0), in line with a simple SIR system, higher transmission rates from the free-living bacterial route (*β*) always burn a significant portion of the population before reaching peak prevalence, leading to a sharp rise in infections and leaving fewer susceptible individuals for the overshoot phase. As a result, the ratio of overshoot to attack rate ($\rho_{\text{OA}}$) decreases monotonically as *β* increases in case of σ=0 (see S4 Fig).

**Fig 5:**
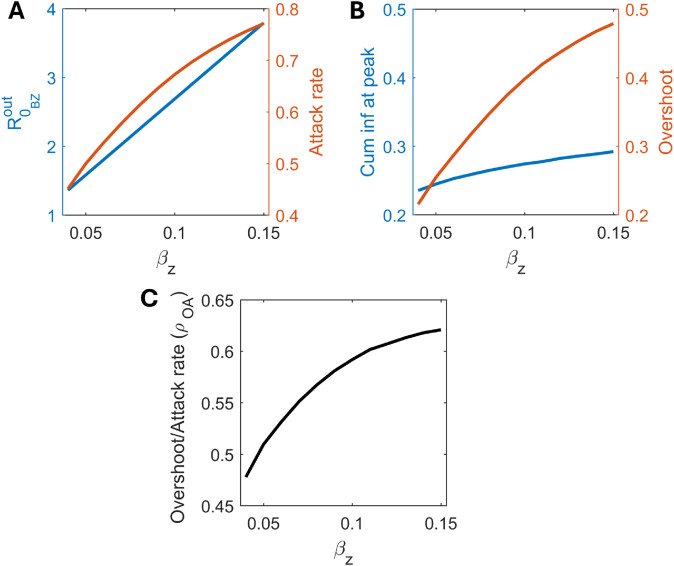
Influence of zooplankton-mediated transmission on epidemic overshoot. Effect of on (A) ${ℛ}_{0_{\text{BZ}}}^{\text{out}}$ and attack rate, (B) cumulative infections at peak (as proportion of the total population) and overshoot, and (C) ratio of overshoot to attack rate ($\rho_{\text{OA}}$). Parameter: σ=0.03.

In presence of bacteria-zooplankton association (σ≠0), increasing transmission via zooplankton ($\beta_{z}$) for a fixed *β* alters the above observations. We find that, along with cumulative infections at the peak and the attack rate, the overshoot also increases substantially across a wide range of $\beta_{z}$ (see Fig 5A and 5B). Interestingly, it appears that the increase in the overshoot due to $\beta_{z}$ profoundly surpasses the rise in cumulative infections at the peak (solid red and blue line Fig 5B). For instance, 50% increase in $\beta_{z}=0.05$ results in 7% and 30% increase in cumulative infections at peak and the overshoot, respectively (Fig 5B). Consequently, the ratio of overshoot to attack rate ($\rho_{\text{OA}}$) increases significantly over a wide range of $\beta_{z}$, which is contrary to what is usually observed in SIR systems (see Fig 5C). This result is particularly interesting, indicating a substantial overshoot in cases where transmission via zooplankton is more pronounced. Therefore, alongside transmission from the free-living bacterial route, even moderate transmission via zooplankton infects a larger portion of the susceptible population in the post-peak phase by sustaining lower-level infections over an extended period before eventually fading out. This underscores that maintenance of control measures during the post-peak phase becomes crucial to prevent additional overshoot. It should be noted that extremely large $\beta_{z}$ values (for which ${ℛ}_{0_{\text{BZ}}}^{\text{out}}$ may not be feasible for cholera (Eq. (3.1))) cause both the overshoot and $\rho_{\text{OA}}$ to decline again (not shown here).

### 3.2. Disease management via water filtration

In the Bengal delta region, numerous studies have documented seasonal fluctuations in *Vibrio* cells attached to zooplankton, with the highest concentrations observed during the early spring and summer, coinciding with the peak zooplankton populations [11,13,14,60]. We note that our model, by explicitly considering the various ecological time scales involved, presents an opportunity to incorporate seasonality and its connection to disease dynamics and potential control measures. To qualitatively mimic the emergence of cholera infections preceded by biannual plankton blooms, we consider the phytoplankton carrying capacity (*K*) to be a periodic function given by


$$K(t)=K_{0}(1+d\sin\left(2\pi t/p\right)).$$

Here *K*_0_ stands for the baseline phytoplankton carrying capacity, while *d* and *p* denote the amplitude and period of oscillation, respectively. This assumption is reasonable as the carrying capacity depends on fluctuations in climatic and environmental factors such as coastal sea surface temperature, nutrient load, salinity, pH, flooding and streamflow [43–45,59,60,63,64]. Note that we ensure *K*(*t*) remains below the critical carrying capacity *K^c^*, beyond which the plankton system no longer shows stable coexistence (see Sect C in S1 Text). In this case, we also consider human demographic and immunity factors (Λ,μ,ω≠0).

The copepod-associated *Vibrio cholerae* cells remains viable even after treatment of household water with chemical disinfectants like alum and chlorination [31]. However, a sari cloth folded four times can remove up to 99% bacteria-associated zooplankton cells, as demonstrated in the laboratory-based study by Huq et al. [65]. Furthermore, an experimental study conducted in Matlab, Bangladesh, by Colwell et al. [66] found that filtration using simple sari and nylon cloths can remove around 90% of bacteria cells attached to copepods. Hence simple filtration of household water can be a more effective method of controlling cholera compared to chemical-based purification. This is particularly relevant in adverse situations including humanitarian crises and climate-driven extreme weather events, where access to clean water is limited, making an inexpensive, easy-to-use, and socially acceptable household water treatment method like filtration essential [3,67]. Additionally, the waning of vaccine-induced immunity, combined with the ongoing critical shortage of oral vaccines (OCV) further reinforces the need for household control measures [4,68].

Filtration is a point-of-use (POU) water purification method, typically applied when collecting water from reservoirs such as lakes and ponds [66,69,70]. By removing planktonic organisms, filtration effectively reduces zooplankton concentration and thus the infectious dose in household water [37,69]. This, in turn, directly impacts the force of infection via the zooplankton-driven transmission route. Other POU measures, such as boiling and household chlorination [71], are not considered here, as our focus is on assessing the impact of controlling infection through the zooplankton-mediated route. To model filtration, we modified the zooplankton-driven force of infection term as follows:

$$FI_{Z}(t)=\left\{ \begin{array}{c} \beta_{z}\frac{\left(1-e_{f})Z_{B}(t\right)}{h_{z}+(1-e_{f})Z_{B}(t)},\text{ during filtration}\\ \beta_{z}\frac{Z_{B}(t)}{h_{z}+Z_{B}(t)}\;\;\;\;\text{ else.} \end{array}\right.$$ (3.2)

Here *e*_*f*_ stands as the filtration efficacy, which quantifies the proportion of dispelled zooplankton cells and depends on the procedure’s accuracy and the mesh size of the used material. For instance, sari cloths appeared to be more effective than nylon nets in removing copepods [66]. Note that we assume an instantaneous effect of filtration on $FI_{Z}(t)$, which is reasonable as the water is typically collected on a daily basis for household use.

An important question is when to initiate filtration and how long the control measure should last. Notably, plankton blooms typically serve as an early warning indicator for cholera outbreaks, with a lag of nearly 8 weeks between plankton blooms and the rise in cholera infections, as observed in the Bengal Delta region [13,24]. So, one can think of two indicators for initiation of filtration: the occurrence of phytoplankton blooms, which are mostly recognizable in water reservoirs, and an increase in cholera infections. Since plankton blooms generally last for about three months, we focus on implementing periodic filtration with same duration. We assess the outcome of filtration by measuring the reduction in the number of infections over a year, compared to the situation without filtration. Implementing filtration measures based on the two indicators implies different initiation times, which may lead to differences in cholera case reduction. We also analyze the impact of filtration initiated at all time points, while maintaining the same duration as above for each case.

**Fig 6:**
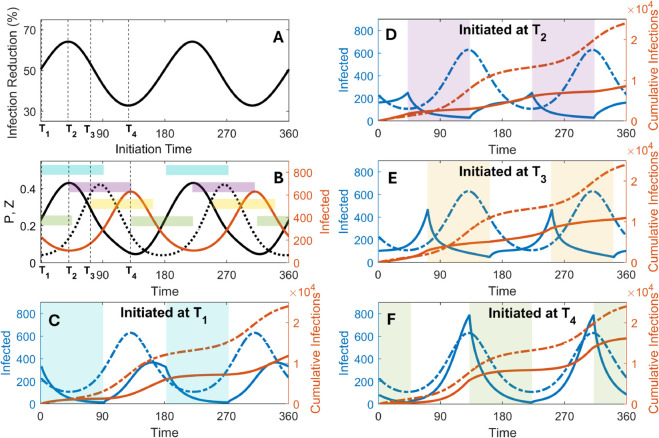
Effect of 90-day periodic filtration on reducing cholera infections linked to seasonal biannual plankton blooms. (A) the percentage of infection reduction (solid line) over a year for different initiation timings. Here *T*_1_-*T*_4_ represents four distinct initiation timings. (B) Illustrates potential indicators for initiating filtration, based on increase in phytoplankton density over time (solid black line) and subsequent increases in infections (red line) along with the zooplankton abundance (dotted black line). The scenario when filtration is initiated at *T*_1_ following the phytoplankton bloom (C), at *T*_2_ when zooplankton abundance is increasing (D) (best-case scenario), at *T*_3_ in response to an increasing trend in infections (E) and at *T*_4_ when delayed until near the infection peak (F) (worst-case scenario). In (C)-(F), active (blue) and cumulative (red) infections are shown for both filtered (solid lines) and unfiltered (dashed lines) scenarios. The shaded regions indicate the filtration periods. Here we consider filtration with 80% efficacy (i.e. *e*_*f*_ = 0.8) and *K*(*t*) is parameterized by *d* = 0.8 and *K*_0_ = 0.27.

The overall effect of filtration on reducing cholera infection at different initiation timings is illustrated in Fig 6. The solid line in Fig 6A indicates the percentage reduction in cholera infections associated with different filtration initiation timings (dashed vertical lines). Fig 6B depicts two filtration indicators: the phytoplankton density (solid black line) and the subsequent rise in infections (red line), alongside zooplankton abundance (dotted black line). Four different filtration initiation timings, *T*_1_-*T*_4_ are considered among which *T*_1_ and *T*_3_ follow the former and the latter indicator, respectively (Fig 6A and 6B). Additionally, the active and cumulative infections under filtration corresponding to timings *T*_1_-*T*_4_, along with the unfiltered scenario, are shown in Fig 6C–6F, respectively. We observe that, when filtration is employed with an efficacy of 80% (i.e. *e*_*f*_ = 0.8), the reduction in cholera infections varies from approximately 32% to 65%, depending on the timing of initiation (Fig 6A). Filtration initiated at *T*_1_ following a phytoplankton bloom reduces around 50% infections (Fig 6C), which is slightly less effective than the 54% reduction observed when filtration is initiated at *T*_3_ in response to increasing cholera infections (Fig 6E).

The above results can be explained by tracking the formation and ingestion of *Z*_*B*_ in our simulations. Filtration initiated at *T*_1_ ends when *Z* abundance is close to its peak, leading to considerable formation of *Z*_*B*_ which are then ingested (Figs 6C and S5A). On the other hand, when filtration is initiated at *T*_3_, in spite of significant *Z*_*B*_ formation, it can lead to relatively less ingestion during the high abundance period (Figs 6E and S5C). Filtration initiated at *T*_2_, which continues during periods of high *Z* abundance, effectively restricts both formation and ingestion of *Z*_*B*_, appears to be the best-case scenario (Figs 6D and S5B). Hence, in this case, infections remain relatively low throughout the year (nearly 65% reduction in Fig 6(D)). This indicates that initiating filtration during rising zooplankton abundance (*T*_2_), rather than in response to rising infections (*T*_3_), leads to comparatively greater reduction in infection. The worst-case scenario arises from delayed filtration initiated near the infection peak at *T*_4_, which fails to restrict both formation and ingestion of *Z*_*B*_ (S5D Fig). In this case, a rapid peak, even larger than the uncontrolled one arises although with a reduced total infection over the year (nearly 32% reduction in Fig 6F). Moreover, infection reduction increases with filtration efficacy, with incremental reduction becoming more pronounced at higher efficacy levels (see S6 Fig and Sect E in S1 Text). Overall, these observations underscore the importance of timely and accurate filtration practices for reducing cholera infections.

## 4. Discussion

Cholera remains a reemerging disease of poverty for nearly 2 billion people in over 50 countries who have inadequate access to safe water and poor sanitation infrastructure [7]. Despite the well-documented correlation between plankton blooms and cholera outbreaks over the past decades, the impact of plankton ecology on cholera dynamics has not yet been fully explored from a mathematical modeling perspective. To this aim, we integrate phytoplankton-zooplankton interactions into the classical human-bacteria (SIRB) cholera model through the ecological commensal association between *V. cholerae* and zooplankton. This could lead different dynamics of cholera outbreak which has remain unexplored until now. For instance, an outbreak that might initially decay without the bacteria-zooplankton association can grow when the transmission via zooplankton is involved. On the other hand, this association can also lead to decline in initial infections which would have grown otherwise. Additionally, there will be scenarios where transmission via zooplankton increases the spread of the disease, i.e., ${ℛ}_{0_{\text{BZ}}}^{\text{out}}$ (Fig 2).

The basic reproduction number (${ℛ}_{0_{\text{BZ}}}^{\text{out}}$) alone may not provide complete picture about outbreak trajectories or disease severity. The relative contribution of transmission routes, influenced by ecological, environmental, and climatic factors, plays a significant role in determining the progression and impact of an outbreak (Fig 3). Although the zooplankton route is responsible for fewer infections compared to the free-living bacterial route, the outbreak persists longer in the presence of bacteria-associated zooplankton cells. The colonization of zooplankton by *V. cholerae* cells prior to human exposure makes the zooplankton route a delayed transmission pathway. This is consistent with the the results of [72]. For a fixed reproduction number, the dominant free-living bacterial route drives rapid outbreak growth with a higher peak. In contrast, even moderate transmission via the zooplankton route results in a comparatively slower outbreak progression with a reduced peak. However, this leads to a higher herd immunity threshold, larger final size, and longer outbreak duration. This finding aligns with the contrasting epidemiological patterns of cholera: the Ganges delta region experiences consistent, longer outbreaks involving reservoir transmission, while the African region often reports shorter, sporadic outbreaks without reservoir transmission, as noted by Sack et al. [73]. This result emphasizes the consideration of relative contributions of transmission routes while designing interventions. Also, it underscores the importance of maintaining control measures despite a slower initial growth of the outbreak, particularly in regions with evidence of *V. cholerae* reservoirs.

Even when the basic reproduction number (${ℛ}_{0_{\text{BZ}}}^{\text{out}}$) increases due to zooplankton-mediated transmission, we can observe both an early or a delayed peak, depending on the different rates of the bacteria-zooplankton association. While the former is the usual expectation, the latter is unintuitive. However, in both cases, a larger outbreak size is reached due to prolonged infections at lower levels during the post-peak phase (Fig 4). This highlights the role of environmental reservoirs in prompting the inter-epidemic persistence of pathogens, as noted in previous studies [15,18,73]. In fact, the increase in overshoot may surpass the rise in cumulative infections at the peak when the transmission rate via zooplankton increases (Fig 5). This contrasts with classical SIR systems, in which the ratio of overshoot to attack rate declines monotonically as transmission increases [62]. This finding highlights the possibility of substantial overshoot in regions with the evidence of *V. cholerae* reservoirs, such as the Ganges delta region, and underscores the need to maintain control measures during the post-peak period. Relaxing control measures beyond the peak of infections, even after achieving herd immunity, could potentially lead to additional overshoot.

Lastly, we also study disease management via practical control measure such as water filtration, which has previously been deemed effective to reduce cholera in endemic regions like Matlab, Bangladesh [66]. Our findings indicate that the timing of implementing such a control measure can be a key to a substantial (approximately 32−65%) reduction in cholera infections (Fig 6). Initiating filtration during rising zooplankton abundance yields the greatest infection reductions, underscoring the importance of ecological cues over prevalence data for timely cholera control. Seasonal plankton blooms, which precede cholera outbreaks by approximately 8 weeks, also offer another critical window for intervention. Phytoplankton can be monitored using remote sensing tools like chlorophyll-a concentration, while zooplankton trends can be inferred from seasonal historical data or localized sampling. Moreover, for real-world applications, the model can be calibrated using time-series data on cholera incidence and plankton abundance. This would improve forecast accuracy, and can guide the timing of low-cost, point-of-use interventions like water filtration. Furthermore, in this study, we focused exclusively on filtration as a control measure because of its unique ability to target the zooplankton-mediated transmission pathway. However, future studies could investigate its combined effect with other intervention strategies such as implementing safe water, basic sanitation and hygiene (WASH) and providing oral vaccination. In fact, bloom forecasts could also guide reactive vaccination campaigns, targeted distribution of filtration kits, water resource management and other POU measures such as boiling or household chlorination. Hence, incorporating ecological monitoring into cholera preparedness frameworks could enable efficient and seasonally adaptive intervention strategies. Therefore, although environmental reservoirs of *V. cholera* in coastal ecosystems complicate eradication, their associated ecology can also provide an opportunity to control cholera endemicity through timely and efficient interventions.

Increased climate variability with ongoing global change and subsequent extreme weather events can influence pathogen ecology, thereby highlighting the need to integrate the same while studying disease dynamics [74]. Our model takes a first step in this direction in the context of cholera and provides qualitative insights into transmission dynamics which could be useful to inform public health policies. Despite the usefulness of our model, it has few limitations. While some studies indicate that *V. cholerae* can also associate with phytoplankton [10,75], we choose to neglect this aspect in our study. This assumption can also be justified in line with the empirical studies [13,22], where an increase in *V. cholerae* concentration has reportedly corresponded to a significant decrease in phytoplankton abundance driven by higher nutrient availability and reduced level of antibacterial metabolites. Additionally, we do not take into account the growth of *V. cholerae* [12,18] and the increased pathogen virulence [15,76] while attaching to zooplankton. Incorporating these ecological and epidemiological aspects in future cholera research could enhance our understanding of disease dynamics. The effect of other environmental factors such as nutrient cycling, temperature-dependent colonization coefficients could also add more realism to the model and could be investigated individually or in combination. The need of the hour is to shift the focus toward integrating the ecology of pathogens and their response to changing environment while predicting disease outbreaks. This could be integrated with socio-economic factors such as variability in sanitation infrastructure and human mobility affecting access to clean water and the uptake of interventions like vaccination or filtration, to provide a more holistic framework for cholera prevention.

## Supporting information

S1 Text Supplementary material. This file includes parameter definitions, proofs of positive invariance and boundedness, analysis of the phytoplankton–zooplankton model, outbreak dynamics (including sensitivity and epidemic growth analysis), and long-term dynamics (basic reproduction number and filtration efficacy impacts).
(PDF)

S1 FigInfluence of zooplankton death rate on the basic reproduction threshold.Zooplankton death rate (*d*_*z*_) increases the slope of the ${ℛ}_{0_{\text{BZ}}}^{\text{out}}={ℛ}_{0}^{\text{out}}$ line and thereby decreasing region 1. Here, for the solid line, *d*_*z*_ = 0.06, and for the dashed line, *d*_*z*_ = 0.1.(TIF)

S2 FigInfluence of *σ* and $\beta_{z}$ on the epidemic growth rate.Initial epidemic growth rate (EGR) with respect to the *Vibrio*-zooplankton association rate (*σ*) for different zooplankton-mediated transmission rate ($\beta_{z}$) within region 1 in Fig 2.(TIF)

S3 FigPost-peak maintenance of low-level infections.Increased zooplankton-mediated transmission ($\beta_{z}$) can have potentially large negative impacts on human health under a fixed bacteria-zooplankton association rate (σ=0.03) within region 1 in Fig 2. It shortens the time to peak infection, increases the peak size, and elevates lower-level maintenance of infections during the post-peak period.(TIF)

S4 FigEffect of free-living bacterial transmission on epidemic dynamics of the classical SIRB model.Effect of transmission rate via the free-living bacterial route (*β*) for the classical SIRB model on (A) ${ℛ}_{0}^{\text{out}}$ and attack rate, (B) cumulative infections at peak (as a proportion of the total population) and epidemic overshoot, (C) the ratio of overshoot to attack rate ($\rho_{\text{OA}}$), and (D) peak timing and epidemic duration in the absence of bacteria-zooplankton association (σ=0). Intuitively, both ${ℛ}_{0}^{\text{out}}$ and attack rate increase with *β*. While *β* increases cumulative infections at peak, there exists an upper bound on the overshoot. The ratio $\rho_{\text{ OA}}$ decreases monotonically with increasing *β* due to the availability of fewer susceptible individuals for the overshoot (post-peak) phase. Both peak timing and epidemic duration consistently decrease as *β* increases.(TIF)

S5 Fig *Z*_*B*_ formation governed by available bacterial cells during zooplankton abundance. The formation of *Z*_*B*_ (solid and dashed blue) depends on the density of *B* cells (solid and dashed red) in the water column during periods of *Z* abundance (dotted blue line) in both unfiltered (dashed) and filtered (solid) scenarios when filtration is initiated at *T*_1_-*T*_4_. The shaded regions indicate the filtration periods. Filtration reduces cholera infections in two ways: first, by restricting *Z*_*B*_ formation through the reduction of *B* cell concentration during the periods *Z* abundance; and second, if *Z*_*B*_ formation cannot be avoided, by preventing its ingestion.
(TIF)

S6 Fig Impact of filtration efficacy on cholera infection reduction. Cholera infection reduction (%) over a year under varying filtration efficacy (*e*_*f*_). The solid line denotes the maximum reduction and the dotted line denotes the minimum, calculated across all possible filtration initiation timings.
(TIF)
