# Supplementary material for: Coupling plankton and cholera dynamics: Insights into outbreak prediction and practical disease management
Source: PLoS Comput Biol. 2025 Sep 29;21(9):e1013523. doi: 10.1371/journal.pcbi.1013523 (PMC12507262; doi:10.1371/journal.pcbi.1013523)
Supplement: S1 Text — This file includes parameter definitions, proofs of positive invariance and boundedness, analysis of the phytoplankton–zooplankton model, outbreak dynamics (including sensitivity and epidemic growth analysis), and long-term dynamics (basic reproduction number and filtration efficacy impacts). (PDF) [file pcbi.1013523.s001.pdf]

S1 Text - Supplementary material for:

Coupling plankton and cholera dynamics: insights into outbreak prediction and practical disease management

Biplab Maity, Swarnendu Banerjee*, Abhishek Senapati,
Jon Pitchford, Joydev Chattopadhyay
*s.banerjee2@uva.nl

September 20, 2025

A. Parameter definitions and calibration

The model summarized by Eq. (2.1) comprises a complex dynamical system involving organisms that vary across many orders of magnitude in size and have similarly diverse time scales associated with their population dynamics. It is important, therefore, to be precise in defining the values and units of all the model parameters under consideration (see Table 1). As the likelihood of exposure to zooplankton-contaminated water is lower than that of bacteria-contaminated water, we assume the transmission rate via zooplankton (β_z) is less than or equal to the transmission rate via free-living bacteria (β). Moreover, we consider the mean dry weight of zooplankton to be approximately 2 μg (Dumont et al., 1975), with the smaller size copepods being more likely to pass through unfiltered water. A single copepod carries around 10^5 *V. cholerae* cells (De Magny et al., 2008; Colwell and Spira, 1992; Colwell et al., 1996), resulting in a colonization coefficient, c, for the bacteria-zooplankton association of $10^5/0.002 = 5 \times 10^7$ cells/(mg dw). Furthermore, the free-living bacterial transmission reaches half of its maximum at the concentration $h_b = 10^9$ *V. cholerae* cells/L (Hartley et al., 2005). So, the half-saturation constant for transmission via zooplankton, h_z, becomes $10^9/(5 \times 10^7) = 20$ (mg dw)/L. Notably, the parameter h_z is inversely proportional to the bacterial colonization coefficient c. Additionally, in line with

the empirical study (Lipp et al., 2002), we assume that bacteria attach to zooplankton at a relatively lower concentration than h_b, with the maximum attachment rate occurring at a concentration that is 1/100th of h_b. This gives the half-saturation constant for B-Z association $h_m = 10^7$ cells/L.

B. Positive Invariance and boundedness

Lemma B.1. *The solutions (S(t),I(t),R(t),B(t),Z(t),P(t)) of the system (2.1) are uniformly and ultimately bounded on*
$\Omega = \Big\{(S, I, R, B, Z_B, Z_F, P) \in \mathbb{R}^7_+ |\ 0 \le S, I, R \le \dfrac{\Lambda}{\mu}, 0 \le B \le \dfrac{\xi\Lambda}{\mu d_b}, 0 \le Z + \eta P \le \dfrac{K\eta(r_p+\alpha)^2}{4r_p\alpha}\Big\}$.

Proof. Model (2.1) can be expressed as the form

$$\frac{dX}{dt} = A(X(t)), \quad X(0) = X_0 \ge 0,$$

where $X = [S, I, R, B, Z_B, Z_F, P]^T$ and $A(X(t)) = (A_1(X), A_2(X), \ldots, A_7(X))^T$. Now, we have

$$\frac{dS}{dt}|_{S=0} = \Lambda + \omega R \ge 0, \quad \frac{dI}{dt}|_{I=0} = \frac{\beta SB}{h_b + B} + \frac{\beta S Z_B}{h_z + Z_B} \ge 0,$$

$$\frac{dR}{dt}|_{R=0} = \gamma I \ge 0, \quad \frac{dB}{dt}|_{B=0} = \xi I \ge 0, \quad \frac{dZ_B}{dt}|_{Z_B=0} = \sigma\frac{BZ_F}{h_m + B} \ge 0,$$

$$\frac{dZ_F}{dt}|_{Z_F=0} = \eta\frac{\alpha P Z_B}{h_p + P} \ge 0, \quad \frac{dP}{dt}|_{P=0} = 0.$$

Based on the lemma from Yang et al. (1996), it follows that $\mathbb{R}^7_+$ is an invariant set. Consequently, any trajectory of system (2.1) that originates in $\mathbb{R}^7_+$ will remain within this domain for all time.

Let $N(t) = S(t) + I(t) + R(t)$ be the total human population at any given instant of time t. Adding first three equations of the system (2.1) we get, $\dfrac{dN}{dt} = \Lambda - \delta I - \mu N$. This implies that, $\dfrac{dN}{dt} \le \Lambda - \mu N$. Therefore by standard comparison theorem, there exists $t_1 \ge 0$ such that $N(t) \le \dfrac{\Lambda}{\mu}$, for all $t \ge t_1$.

Now from the fourth equation of system (2.1), $\dfrac{dB}{dt} \le \xi I - d_b B$. Again by comparison theorem, there exists $t_2 \ge 0$ such that $B(t) \le \dfrac{\xi\Lambda}{\mu d_b}$ for all $t \ge t_2$.

To show the boundedness of the remaining three compartments, let $M = Z + \eta P = Z_B + Z_F + \eta P$.
Then, $\frac{dM}{dt} = -d_z Z_B + \eta r_p P(1 - \frac{P}{K}) - d_z Z_F$.
Now let $\alpha > 0$. Then $\frac{dM}{dt} + \alpha M = \eta r_p P(1 - \frac{P}{K}) + \alpha\eta P + (\alpha - d_z)Z_F + (\alpha - d_z)Z_B$. We choose α such that $\alpha < d_z$. Then $\frac{dM}{dt} + \alpha M \leq \max\Big\{\eta r_p P(1 - \frac{P}{K}) + \alpha\eta P\Big\} = \frac{K\eta(r_p + \alpha)^2}{4r_p}$. Again by comparison theorem, there exists $t_3 \geq 0$ such that $M(t) \leq \frac{K\eta(r_p + \alpha)^2}{4r_p\alpha}$, for all $t \geq t_3$. □

C. The phytoplankton-zooplankton model

Considering the total zooplankton density as $Z(t) = Z_F(t) + Z_B(t)$, from the last three equations of system (2.1), we have the phytoplankton-zooplankton equation as

$$\begin{aligned} \frac{dZ}{dt} &= \eta\frac{\alpha PZ}{h_p + P} - d_z Z, \\ \frac{dP}{dt} &= r_p P(1 - \frac{P}{K}) - \frac{\alpha PZ}{h_p + P}. \end{aligned} \tag{C.1}$$

Here, the independence of system (C.1) from the human-bacteria (SIRB) dynamics reflects the commensal interaction between *Vibrio cholerae* and zooplankton. The system (C.1) has three equilibrium solutions: trivial $S_0 = (0, 0)$, zooplankton-free $S_1 = (0, K)$ and zooplankton-phytoplankton co-existence $S^* = (Z^*, P^*)$ equilibrium. Where the P^* and Z^* are given by the solution of the following two equations:

$$\eta\frac{\alpha P^* Z^*}{h_p + P^*} - d_z Z^* = 0 \quad \text{and} \quad r_p P^*(1 - \frac{P^*}{K}) - \frac{\alpha P^* Z^*}{h_p + P^*} = 0.$$

Solving the above equations we get,

$$P^* = \frac{d_z h_p}{\eta\alpha - d_z}, \quad Z^* = \frac{r_p}{\alpha}(1 - \frac{P^*}{K})(h_p + P^*) \tag{C.2}$$

Here, $C_P = \frac{\eta\alpha}{d_z} > 1$ is the necessary condition for the existence of positive phytoplankton density. Also, $C_Z = \frac{KC_P}{(h_p + K)} > 1$ with $C_P > 1$ is the necessary condition for co-existence equilibrium density. Now, the local stability of the equilibrium solutions for the system (C.1) follows the lemma below.

Lemma C.1. *The trivial equilibrium solution $S_0 = (0,0)$ of the system (C.1) always exists and is unstable. Zooplankton-free equilibrium solution $S_1 = (0, K)$, which exists and stable when $C_Z < 1$ with $C_P > 1$ and unstable for $C_Z > 1$. Also, the co-existence equilibrium solution $S^* = (Z^*, P^*)$ exists when $C_Z, C_P > 1$. Moreover, for $C_Z, C_P > 1$, there exists a critical value of K^c,*

$$K^c = \frac{h_p(\eta\alpha + d_z)}{(\eta\alpha - d_z)} \tag{C.3}$$

such that the co-existence equilibrium solution S^* is
(i) asymptotically stable when $K < K^c$,
(ii) center when $K = K^c$,
(iii) unstable, associated with the appearance of bounded periodic solutions with initial amplitude and period of $\exp\left(\frac{r_p}{2C_Z} - \frac{r_p h_p}{K(C_P-1)}\right)$ *and* $4\pi/\sqrt{4r_p d_z\big(1-\frac{1}{C_Z}\big) - r_p^2\Big(\frac{1}{C_Z} - \frac{2r_p h_p}{K(C_P-1)}\Big)^2}$*, respectively for $K > K^c$.*

Note that K^c represents the critical phytoplankton carrying capacity beyond which the plankton system (C.1) no longer shows stable coexistence.

D. Outbreak dynamics

Condition for initial outbreak growth

The basic characteristics of an outbreak at an initial stage can be inferred from the sign of $dI/dt|_{t=0}$. The outbreak will initially grow, remain stationary, or decay depending on whether $dI/dt|_{t=0}$ is greater than, equal to, or less than zero, respectively. From the 2nd equation of model (2.1), we have the necessary condition for the initial growth of an outbreak as

$$\frac{\beta S_0 B_0}{h_b + B_0} + \frac{\beta_z S_0 Z_{B_0}}{h_z + Z_{B_0}} > (\gamma + \delta) I_0. \tag{D.1}$$

Here $N_0 = S_0 + I_0$ is the initial human population size, where S_0 and I_0 represent the initial susceptible and infected population, respectively. From the 4th & 5th equation of model (2.1), the initial growth in B, Z_B cells implies $dB/dt > 0$, $dZ_B/dt > 0$, which gives

$$\xi I_0 - d_b B_0 - c\sigma\frac{B_0 Z_F^*}{h_m + B_0} > 0 \quad \text{and} \quad \sigma\frac{B_0 Z_F^*}{h_m + B_0} - d_z Z_{B_0} > 0.$$

After simplification, we have

$$I_0 > \frac{d_b B_0}{\xi} + c\sigma \frac{B_0 Z_F^*}{\xi(h_m + B_0)} \quad \text{and} \quad Z_{B_0} < \sigma \frac{B_0 Z_F^*}{d_z(h_m + B_0)}. \tag{D.2}$$

Using Eq. (D.2), from Eq. (D.1), we have

$$\frac{\beta S_0 B_0}{h_b + B_0} + \frac{\beta_z S_0}{h_z + Z_{B_0}} \sigma \frac{B_0 Z_F^*}{d_z(h_m + B_0)} > \frac{(\gamma + \delta)}{\xi} \Big(d_b B_0 + c\sigma \frac{B_0 Z_F^*}{h_m + B_0} \Big)$$

$$\text{or,} \quad \frac{\xi S_0}{(\gamma + \delta)} \left(\frac{\beta}{h_b + B_0} + \frac{\beta_z}{h_z + Z_{B_0}} \sigma \frac{Z_F^*}{d_z(h_m + B_0)} \right) > \Big(d_b + c\sigma \frac{Z_F^*}{h_m + B_0} \Big).$$

Now, at $t = 0$, from above, $S_0 \approx N_0$ and $B_0, Z_{B_0} \approx 0, Z_{F_0} \approx Z^*$ give

$$\frac{\xi N_0}{(\gamma + \delta)} \left[\frac{\beta}{h_b} + \frac{\beta_z}{h_z} \sigma \frac{Z^*}{d_z h_m} \right] > \Big(d_b + c\sigma \frac{Z^*}{h_m} \Big),$$

$$\textit{or,} \quad \frac{\xi N_0}{(\gamma + \delta)\Big(d_b + c\sigma \frac{Z^*}{h_m} \Big)} \left[\frac{\beta}{h_b} + \frac{\beta_z}{h_z} \sigma \frac{Z^*}{d_z h_m} \right] > 1, \tag{D.3}$$

$$\textit{or,} \quad \mathcal{R}_{0_{\mathrm{BZ}}}^{\mathrm{out}} = \mathcal{R}_{0_B}^{\mathrm{out}} + \mathcal{R}_{0_Z}^{\mathrm{out}} > 1$$

The outbreak initially remains stationary or in a decaying state and subsequently decays whenever $\mathcal{R}_{0_{\mathrm{BZ}}}^{\mathrm{out}} \leq 1$. Here, $\dfrac{\xi}{(\delta + \gamma)}$ represents the average amount of pathogen shed by an infected person throughout their infectious period.

Derivation of β_z on $\mathcal{R}_{0_{\mathrm{BZ}}}^{\mathrm{out}} = 1$ and $\mathcal{R}_{0_{\mathrm{BZ}}}^{\mathrm{out}} = \mathcal{R}_0^{\mathrm{out}}$ line

From Eq. (D.3), we have the critical zooplankton-mediated transmission rate, $\beta_z^{c_1}$, sufficient to initiate an outbreak ($\mathcal{R}_{0_{\mathrm{BZ}}}^{\mathrm{out}} > 1$) for each specific β, as given by:

$$\beta_z^{c_1} = \left(\frac{\Big(d_b + \dfrac{c\sigma Z^*}{h_m} \Big)(\delta + \gamma)}{\xi N_0} - \frac{\beta}{h_b} \right) \frac{h_m h_z d_z}{\sigma Z^*} \tag{D.4}$$

Depending on the bacteria-zooplankton (B-Z) association rate (σ) and the transmission rate via zooplankton (β_z), $\mathcal{R}_{0_{\mathrm{BZ}}}^{\mathrm{out}}$ can be lower or higher than

$\mathcal{R}_0^{\text{out}}(=\mathcal{R}_{0_{\text{BZ}}}^{\text{out}}|_{\sigma=0})$.
Now, on the $\mathcal{R}_{0_{\text{BZ}}}^{\text{out}}=\mathcal{R}_0^{\text{out}}$ line, we have

$$\mathcal{R}_{0_{\text{BZ}}}^{\text{out}}=\mathcal{R}_0^{\text{out}}$$

$$\text{or,}\quad \frac{\xi N_0}{(\gamma+\delta)\Big(d_b+c\sigma\frac{Z^*}{h_m}\Big)}\left[\frac{\beta}{h_b}+\frac{\beta_z}{h_z}\sigma\frac{Z^*}{d_zh_m}\right]=\frac{\xi N_0}{d_b(\gamma+\delta)}\frac{\beta}{h_b}$$

$$\text{or,}\quad \frac{1}{\Big(d_b+c\sigma\frac{Z^*}{h_m}\Big)}\frac{\beta_z}{h_z}\sigma\frac{Z^*}{d_zh_m}=\frac{\beta}{h_b}\left[\frac{1}{d_b}-\frac{1}{\Big(d_b+c\sigma\frac{Z^*}{h_m}\Big)}\right]$$

$$\text{or,}\quad \frac{1}{\Big(d_b+c\sigma\frac{Z^*}{h_m}\Big)}\left(\frac{\beta_z\sigma Z^*}{h_zd_zh_m}-\frac{\beta c\sigma Z^*}{h_bd_bh_m}\right)=0$$

$$\text{or,}\quad \beta_z=\frac{ch_zd_z}{h_bd_b}\beta=\beta_z^{c_2}.$$

Now, $\dfrac{\partial\beta_z^{c_1}}{\partial\beta}<0$ and $\dfrac{\partial\beta_z^{c_2}}{\partial\beta}>0$ indicate that at an increased β, a lower $\beta_z^{c_1}$ is required for the initial outbreak growth, whereas a higher $\beta_z^{c_2}$ is necessary for $\mathcal{R}_{0_{\text{BZ}}}^{\text{out}}>\mathcal{R}_0^{\text{out}}$.

On the line $\mathcal{R}_0^{\text{out}}=1$ $\Big($i.e., $\beta=\dfrac{(\gamma+\delta)h_bd_b}{\xi N_0}\Big)$, we have

$$\begin{aligned}\beta_z^{c_1}&=\frac{(\gamma+\delta)}{\xi N_0}\left(\left(d_b+\frac{c\sigma Z^*}{h_m}\right)-\frac{\xi N_0}{(\gamma+\delta)}\frac{\beta}{h_b}\right)\frac{h_mh_zd_z}{\sigma Z^*}\\&=\frac{(\gamma+\delta)}{\xi N_0}\left(\left(d_b+\frac{c\sigma Z^*}{h_m}\right)-d_b\right)\frac{h_mh_zd_z}{\sigma Z^*}=\frac{(\gamma+\delta)ch_zd_z}{\xi N_0}.\end{aligned}$$

Also, $\beta_z^{c_2}=\dfrac{ch_zd_z}{h_bd_b}\dfrac{(\gamma+\delta)h_bd_b}{\xi N_0}=\dfrac{(\gamma+\delta)ch_zd_z}{\xi N_0}$.

Since $\beta_z^{c_2}$ satisfies $\mathcal{R}_{0_{\text{BZ}}}^{\text{out}}=\mathcal{R}_0^{\text{out}}$, so we have $\beta_z^{c_1}=\beta_z^{c_2}$ at $\mathcal{R}_0^{\text{out}}=\mathcal{R}_{0_{\text{BZ}}}^{\text{out}}=1$.

Effect of σ on $\mathcal{R}_{0\text{BZ}}^{\text{out}}$ within the (β-β_z) parameter space

Now,

$$\begin{aligned}\frac{\partial \beta_z^{c_1}}{\partial \sigma} &= \frac{h_m h_z d_z}{Z^*}\Big(-\frac{(\gamma+\delta)}{\xi N_0}\frac{d_b}{\sigma^2} + \frac{\beta}{h_b \sigma^2}\Big) \\ &= \frac{h_m h_z d_z}{h_b \sigma^2 Z^*}\Big(\beta - \frac{(\gamma+\delta)h_b d_b}{\xi N_0}\Big) \\ &= \frac{h_m h_z d_z}{h_b \sigma^2 Z^*}\frac{(\gamma+\delta)h_b d_b}{\xi N_0}\Big(\mathcal{R}_0^{\text{out}} - 1\Big)\end{aligned}$$

Thus, a higher σ decreases $\beta_z^{c_1}$ when $\mathcal{R}_0^{\text{out}} < 1$ and increases $\beta_z^{c_1}$ when $\mathcal{R}_0^{\text{out}} > 1$. In other words, σ decreases the slope of the line $\mathcal{R}_{0\text{BZ}}^{\text{out}} = 1$. As a result, regions [6], [3] expand at the expense of [5], [2], respectively, with higher σ (see Fig 2).

Now,

$$\begin{aligned}\frac{\partial \mathcal{R}_{0\text{BZ}}^{\text{out}}}{\partial \sigma} &= \frac{\xi N_0}{(\gamma+\delta)} \frac{\frac{Z^*}{h_m}}{\left(d_b + c\sigma \frac{Z^*}{h_m}\right)^2}\left[\frac{\beta_z d_b}{h_z d_z} - \frac{c\beta}{h_b}\right] \\ &= \frac{\xi N_0}{(\gamma+\delta)} \frac{\frac{Z^*}{h_m}}{\left(d_b + c\sigma \frac{Z^*}{h_m}\right)^2}\frac{d_b}{h_z d_z}\Big(\beta_z - \beta_z^{c_2}\Big) \\ & >, \;=\; \text{or} < 0 \quad \text{iff } \beta_z >, \;=\; \text{or} < \beta_z^{c_2}.\end{aligned}$$

The decrease (within [2], [3], [4]) or increase (within [5], [6], [1]) of $\mathcal{R}_{0\text{BZ}}^{\text{out}}$ with higher σ, depending on whether $\beta_z < \beta_z^{c_2}$ or $\beta_z > \beta_z^{c_2}$, can be observed in Fig 2B.

Sensitivity analysis

We perform a global sensitivity analysis of $\mathcal{R}_{0\text{BZ}}^{\text{out}}$ with respect to β and β_z under both low and high σ, using Latin hypercube sampling (LHS) coupled with partial rank correlation coefficients (PRCCs) (Saltelli et al., 2004; Marino et al., 2008). We draw 1,000 samples for each parameter using the LHS scheme, considering a 20% range of variation around baseline values given in Table 1. Under high σ, β_z shows greater sensitivity to $\mathcal{R}_{0\text{BZ}}^{\text{out}}$ compared to lower σ, while as expected, β remains consistently more sensitive in both cases (see Fig A).

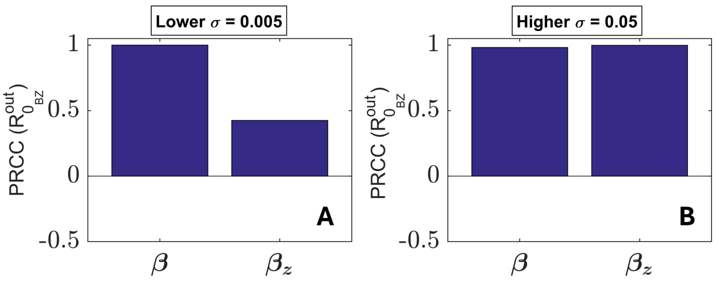

Figure A: Sensitivity indices for $\mathcal{R}_{0_{\mathrm{BZ}}}^{\mathrm{out}}$ w.r.t β, β_z under low σ ($\sigma = 0.005$)(A) and high σ ($\sigma = 0.05$)(B).

Relative contribution of $\mathcal{R}_{0_B}^{\mathrm{out}}$, $\mathcal{R}_{0_Z}^{\mathrm{out}}$ to $\mathcal{R}_{0_{\mathrm{BZ}}}^{\mathrm{out}}$

For a fixed β, to keep $\mathcal{R}_{0_{\mathrm{BZ}}}^{\mathrm{out}}$ same as $\mathcal{R}_0^{\mathrm{out}}$ $\left(\text{i.e., } \beta_z = \dfrac{ch_z d_z}{h_b d_b}\beta\right)$, there exists a unique bacteria-zooplankton association rate (σ) for each pair of relative contributions ($\mathcal{R}_{0_B}^{\mathrm{out}}$, $\mathcal{R}_{0_Z}^{\mathrm{out}}$).
Now,

$$\mathcal{R}_{0_B}^{\mathrm{out}} = \frac{\xi N_0}{(\gamma+\delta)\left(d_b + c\sigma\frac{Z^*}{h_m}\right)}\frac{\beta}{h_b}$$

$$\text{or, } \sigma = \frac{h_m}{cZ^*}\left(\frac{\xi N_0}{(\gamma+\delta)h_b}\frac{\beta}{h_b}\frac{1}{\mathcal{R}_{0_B}^{\mathrm{out}}} - d_b\right)$$

$$= \frac{d_b h_m}{cZ^*}\left(\frac{\mathcal{R}_{0_{\mathrm{BZ}}}^{\mathrm{out}}}{\mathcal{R}_{0_B}^{\mathrm{out}}} - 1\right) = \frac{d_b h_m}{cZ^*}\frac{\mathcal{R}_{0_Z}^{\mathrm{out}}}{\mathcal{R}_{0_B}^{\mathrm{out}}} = \frac{d_b h_m}{cZ^*}\left(\frac{1}{\mathcal{R}_{0_{\mathrm{BZ}}}^{\mathrm{out}}/\mathcal{R}_{0_Z}^{\mathrm{out}} - 1}\right)$$

$$\text{or, } \frac{\mathcal{R}_{0_Z}^{\mathrm{out}}}{\mathcal{R}_{0_{\mathrm{BZ}}}^{\mathrm{out}}} = \frac{c\sigma Z^*}{d_b h_m + c\sigma Z^*}.$$

Epidemic growth rate (EGR)

The initial exponential growth rate (EGR) of an epidemic assesses the early outbreak dynamics and is crucial in inferring the basic reproduction number. The EGR is determined by the dominant eigenvalue of the Jacobian at the disease-free equilibrium point $E^0 = (N_0, 0, 0, 0, 0, Z^*, P^*)$ (Tien and Earn, 2010). For system (2.1), the initial EGR, denoted by λ, can be obtained

from the following equation:

$$x^3 + c_2 x^2 + c_1 x + c_0 = 0$$

Where the coefficients are given by

$$\begin{aligned}
c_2 &= d_b + \frac{c\sigma Z^*}{h_m} + \gamma + \delta + d_z > 0, \\
c_1 &= \Big(\frac{c\sigma Z^*}{h_m} + d_b + d_z\Big)(\gamma + \delta) + \Big(\frac{c\sigma Z^*}{h_m} + d_b\Big)d_z - \frac{N_0 \xi \beta}{h_b}, \\
c_0 &= (\gamma + \delta)\Big(\frac{c\sigma Z^*}{h_m} + d_b\Big)d_z - \frac{N_0 \xi \beta d_z}{h_b} - \frac{N_0 \xi \beta_z}{h_z}\frac{\sigma Z^*}{h_m}, \\
&= (\gamma + \delta)\Big(\frac{c\sigma Z^*}{h_m} + d_b\Big)d_z\Big(1 - \mathcal{R}_{0\mathrm{BZ}}^{\mathrm{out}}\Big).
\end{aligned}$$

For $\mathcal{R}_{0\mathrm{BZ}}^{\mathrm{out}} < 1$, we have $c_0 > 0$ and can also show that $c_1 > 0$. Using the Descartes' rule of signs, we have $\lambda < 0$, which indicates that the outbreak will not initiate with a few initial infections. However, for $\mathcal{R}_{0\mathrm{BZ}}^{\mathrm{out}} > 1$, we get $c_0 < 0$. In this case, regardless of the sign of c_1, we have $\lambda > 0$, implying that the disease has the potential to invade the population.

The impact of increasing σ on the EGR depends on the value of β_z within region $\boxed{1}$ of Fig 2 (see S2 Fig).

Post-peak maintenance of low-level infections

Here, we investigate the impact of varying transmission rates via zooplankton (β_z) on outbreak trajectories, under a fixed bacteria-zooplankton association rate (σ) within $\boxed{1}$ in Fig 2. Zooplankton serves as a reservoir for *Vibrios*, helping to sustain lower-level infections over an extended post-peak phase before finally dying out (see S3 Fig).

E. Long-term dynamics

The basic reproduction number ($\mathcal{R}_{0\mathrm{BZ}}^{l}$)

Using the next-generation matrix approach (Diekmann et al., 2010), we obtain the basic reproduction number ($\mathcal{R}_{0\mathrm{BZ}}^{l}$) in long-term scenario ($\Lambda, \mu, \omega \neq 0$), defined as the average number of secondary infections appearing from an average primary case within an entirely susceptible population.
When $C_Z, C_P > 1$ and $K < K^c$ (see Eq. (C.3) and Lemma C.1), the disease-free equilibrium (DFE) for the model (2.1) is given by $E^0 = (\frac{\Lambda}{\mu}, 0, 0, 0, 0, Z^*, P^*)$.

If $X = (I, B, Z_B)^T$, which are the infected compartments, then we can write $\dfrac{dX}{dt} = (\mathcal{F} + \mathcal{V})X$ from Eq. (2.1). Where,

$$\mathcal{F} = \begin{pmatrix} 0 & \dfrac{\beta h_b S}{(h_b + B)^2} & \dfrac{\beta_z h_z S}{(h_z + Z_B)^2} \\ 0 & 0 & 0 \\ 0 & 0 & 0 \end{pmatrix},$$

$$\mathcal{V} = \begin{pmatrix} -(\gamma + \mu + \delta) & 0 & 0 \\ \xi & -d_b - c\sigma\dfrac{h_m Z_F}{(h_m + B)^2} & 0 \\ 0 & \sigma\dfrac{h_m Z_F}{(h_m + B)^2} & -d_z \end{pmatrix}.$$

This gives,

$$F = \mathcal{F}|_{E^0} = \begin{pmatrix} 0 & \dfrac{\beta\Lambda}{h_b\mu} & \dfrac{\beta_z\Lambda}{h_z\mu} \\ 0 & 0 & 0 \\ 0 & 0 & 0 \end{pmatrix}, \; V = -\mathcal{V}|_{E^0} = \begin{pmatrix} \gamma + \mu + \delta & 0 & 0 \\ -\xi & d_b + \dfrac{c\sigma Z^*}{h_m} & 0 \\ 0 & -\dfrac{\sigma Z^*}{h_m} & d_z \end{pmatrix}.$$

Now,

$$FV^{-1}$$

$$= \begin{pmatrix} 0 & \dfrac{\beta\Lambda}{h_b\mu} & \dfrac{\beta_z\Lambda}{h_z\mu} \\ 0 & 0 & 0 \\ 0 & 0 & 0 \end{pmatrix} \begin{pmatrix} \dfrac{1}{\gamma + \mu + \delta} & 0 & 0 \\ \dfrac{h_m\xi}{(d_b h_m + c\sigma Z^*)(\delta + \gamma + \mu)} & \dfrac{h_m}{d_b h_m + \sigma Z^*} & 0 \\ \dfrac{\xi\sigma Z^*}{d_z(d_b h_m + c\sigma Z^*)(\delta + \gamma + \mu)} & \dfrac{\sigma Z^*}{d_z(d_b h_m + \sigma Z^*)} & \dfrac{1}{d_z} \end{pmatrix}$$

We have the basic reproduction number for the long-term scenario as

$$\begin{aligned} \mathcal{R}^l_{0_{BZ}} &= \rho(FV^{-1}) \\ &= \frac{\xi\Lambda}{\mu(d_b + \dfrac{c\sigma Z^*}{h_m})(\delta + \gamma + \mu)}\left(\frac{\beta}{h_b} + \frac{\sigma Z^*}{d_z h_m}\frac{\beta_z}{h_z}\right) \qquad \text{(E.1)} \\ &= \mathcal{R}^l_{0_B} + \mathcal{R}^l_{0_Z}. \end{aligned}$$

Here, the components $\mathcal{R}^l_{0_B}$ and $\mathcal{R}^l_{0_Z}$ are associated with transmission via the B and Z_B route, respectively.

Impact of filtration efficacy on infection reduction

In order to assess the sensitivity of cholera infection reduction to filtration efficacy (e_f), we varied e_f systematically over a range from 40% to 90%. For each efficacy level, we calculated the maximum (solid line) and minimum (dotted line) infection reduction over a year across all possible filtration initiation timings. As shown in S6 Fig, infection reduction exhibits a nonlinear relationship with efficacy, with incremental reduction becoming more pronounced at higher efficacy levels. Moreover, the difference between minimum and maximum reductions becomes more prominent at greater efficacy values. These findings underscore the importance of timely implementation of filtration practices with high procedural accuracy and appropriate materials.

References

Colwell, R., Brayton, P., Herrington, D., Tall, B., Huq, A., Levine, M., 1996. Viable but non-culturable vibrio cholerae o1 revert to a cultivable state in the human intestine. World Journal of Microbiology and biotechnology 12, 28–31.

Colwell, R.R., Spira, W.M., 1992. The ecology of vibrio cholerae, in: Cholera. Springer, pp. 107–127.

De Magny, G.C., Murtugudde, R., Sapiano, M.R., Nizam, A., Brown, C.W., Busalacchi, A.J., Yunus, M., Nair, G.B., Gil, A.I., Lanata, C.F., et al., 2008. Environmental signatures associated with cholera epidemics. Proceedings of the National Academy of Sciences 105, 17676–17681.

Diekmann, O., Heesterbeek, J.A.P., Roberts, M.G., 2010. The construction of next-generation matrices for compartmental epidemic models. Journal of the royal society interface 7, 873–885.

Dumont, H.J., Van de Velde, I., Dumont, S., 1975. The dry weight estimate of biomass in a selection of cladocera, copepoda and rotifera from the plankton, periphyton and benthos of continental waters. Oecologia 19, 75–97.

Hartley, D., Morris Jr, J., Smith, D., 2005. Hyperinfectivity: A Critical Element in the Ability of v. cholerae to Cause Epidemics? PLoS Med 3, e7.

Lipp, E.K., Huq, A., Colwell, R.R., 2002. Effects of global climate on infectious disease: the cholera model. Clinical microbiology reviews 15, 757–770.

Marino, S., Hogue, I.B., Ray, C.J., Kirschner, D.E., 2008. A methodology for performing global uncertainty and sensitivity analysis in systems biology. J. Theor. Biol. 254, 178–196.

Saltelli, A., Tarantola, S., Campolongo, F., Ratto, M., et al., 2004. Sensitivity analysis in practice: a guide to assessing scientific models. Chichester, England .

Tien, J.H., Earn, D.J., 2010. Multiple transmission pathways and disease dynamics in a waterborne pathogen model. Bulletin of mathematical biology 72, 1506–1533.

Yang, X., Chen, L., Chen, J., 1996. Permanence and positive periodic solution for the single-species nonautonomous delay diffusive models. Computers & Mathematics with Applications 32, 109–116.
