# Supplementary figures and images for: Coupling plankton and cholera dynamics: Insights into outbreak prediction and practical disease management
Source: PLoS Comput Biol. 2025 Sep 29;21(9):e1013523. doi: 10.1371/journal.pcbi.1013523 (PMC12507262; doi:10.1371/journal.pcbi.1013523)

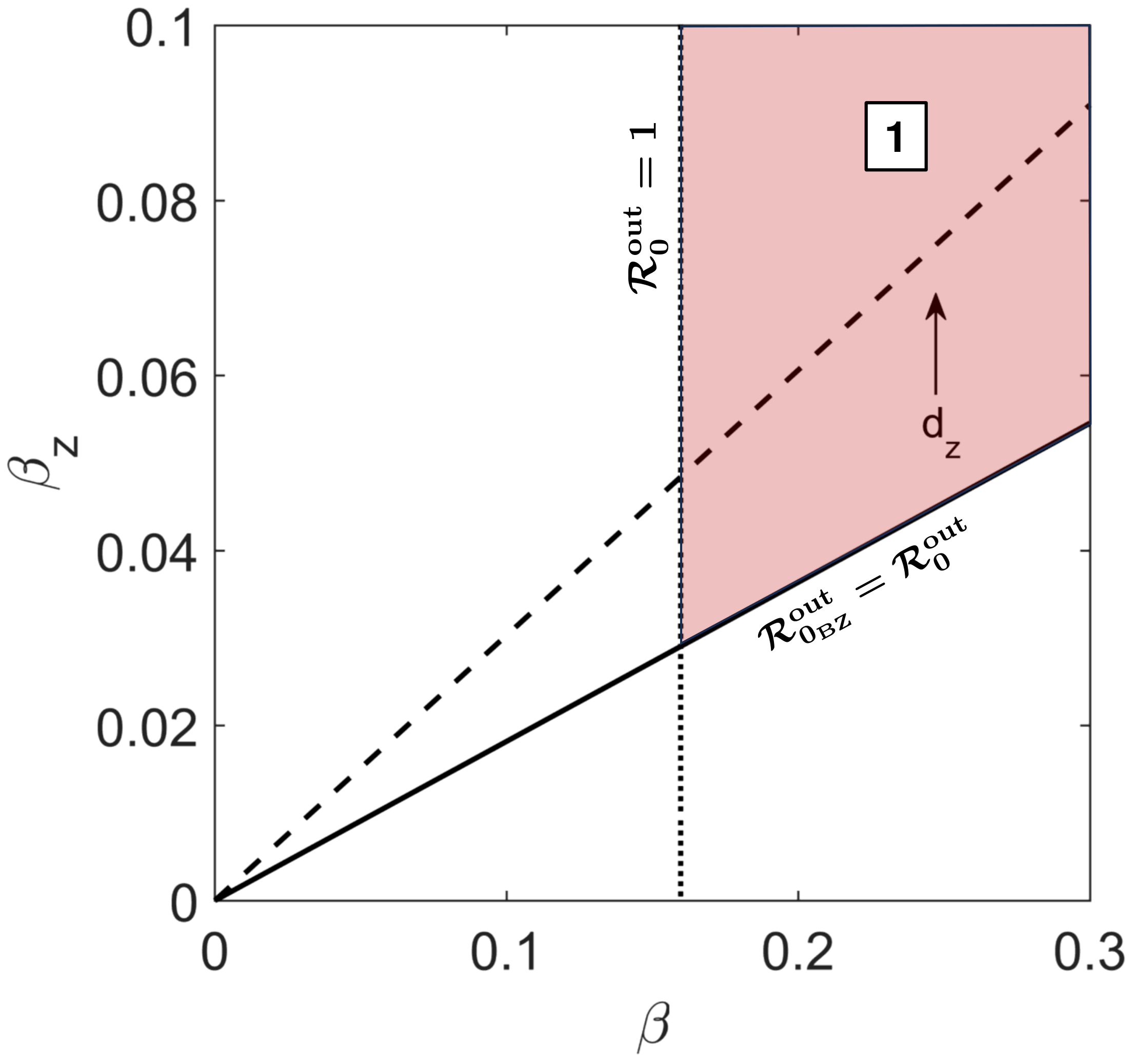

Supplement: S1 Fig — Zooplankton death rate (dz) increases the slope of the ℛ0BZout=ℛ0out line and thereby decreasing region 1. Here, for the solid line, dz = 0.06, and for the dashed line, dz = 0.1. (TIF) [file pcbi.1013523.s002.tif]

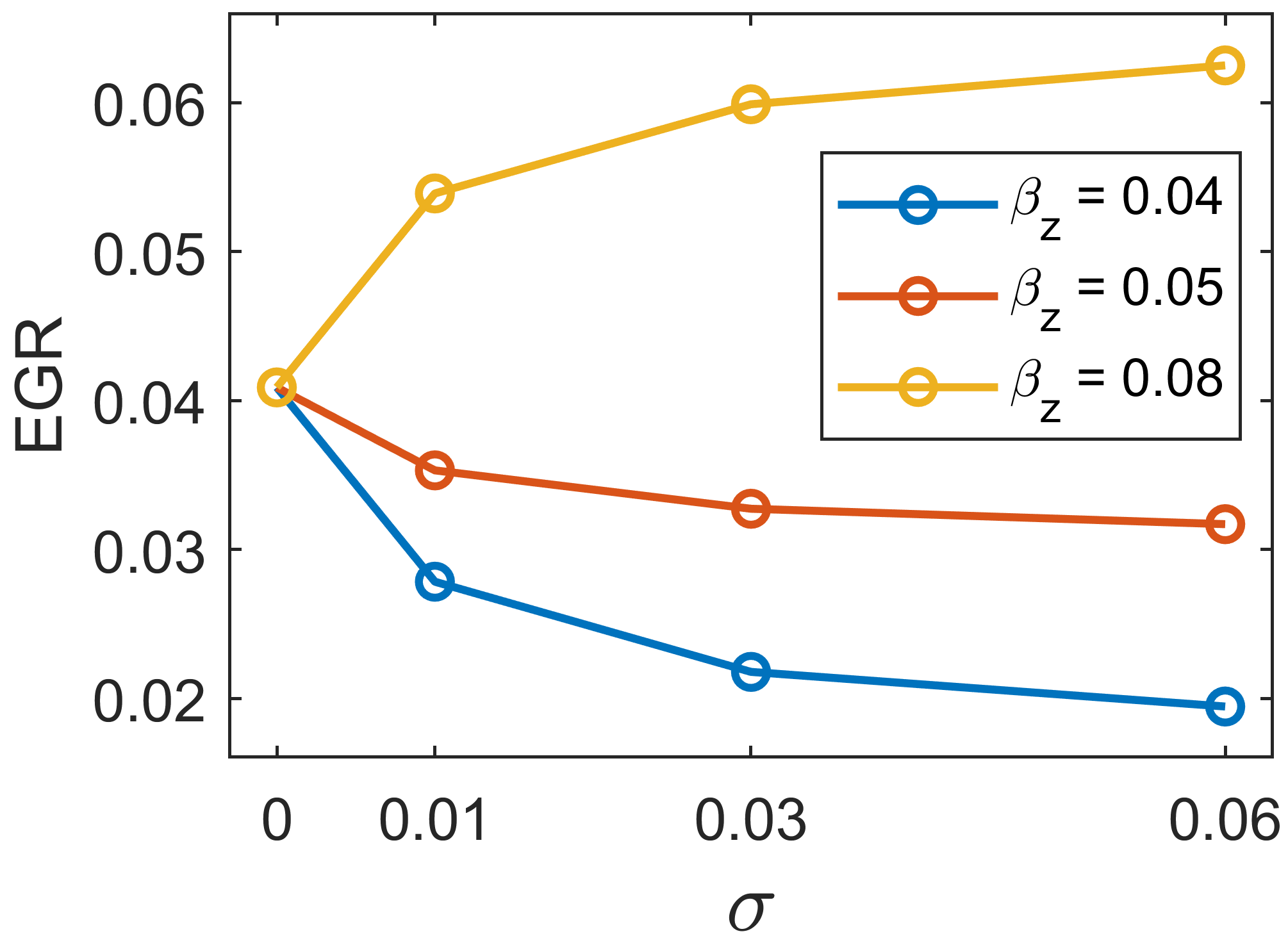

Supplement: S2 Fig — Initial epidemic growth rate (EGR) with respect to the Vibrio-zooplankton association rate (σ) for different zooplankton-mediated transmission rate (βz) within region 1 in Fig 2. (TIF) [file pcbi.1013523.s003.tif]

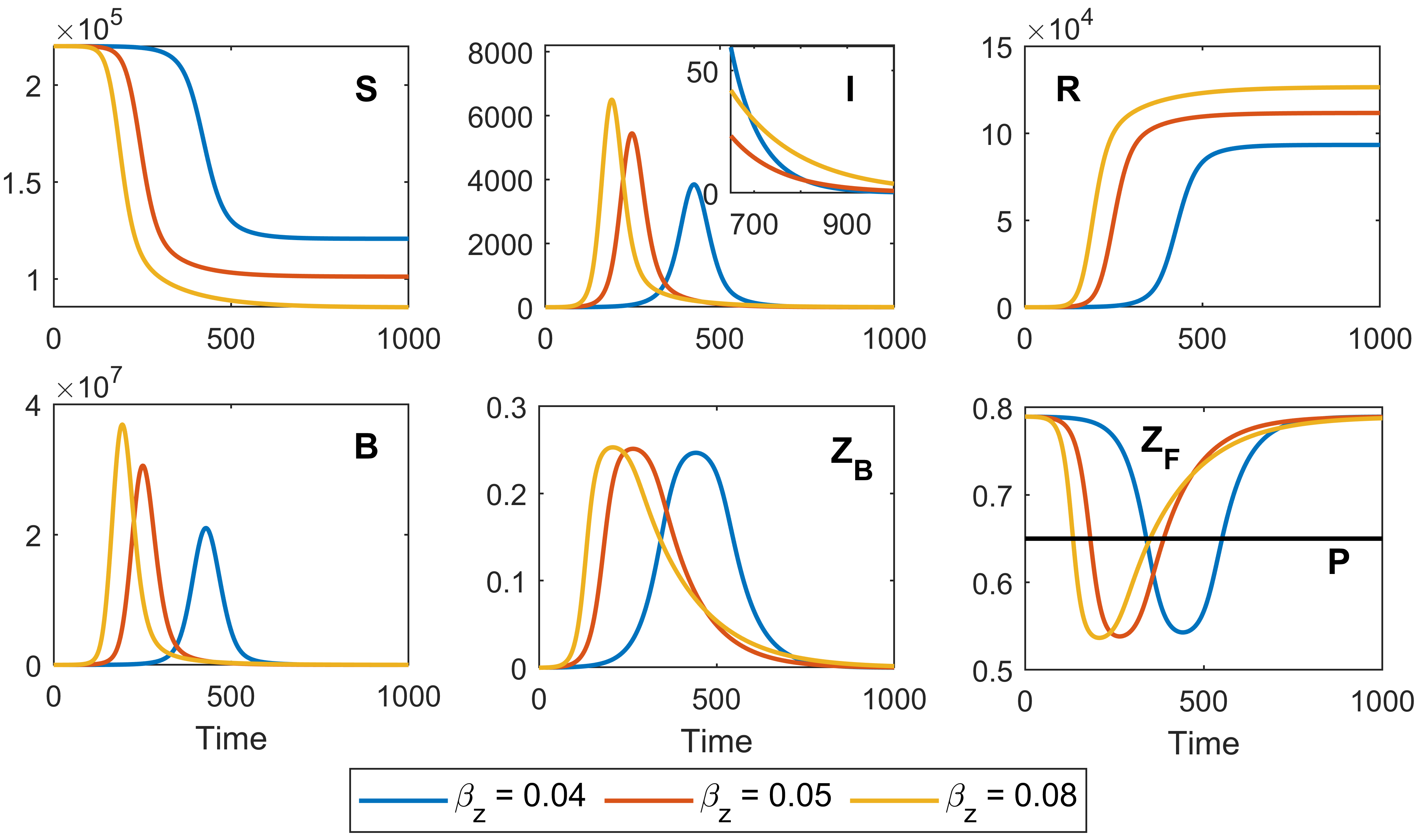

Supplement: S3 Fig — Increased zooplankton-mediated transmission (βz) can have potentially large negative impacts on human health under a fixed bacteria-zooplankton association rate (σ=0.03) within region 1 in Fig 2. It shortens the time to peak infection, increases the peak size, and elevates lower-level maintenance of infections during the post-peak period. (TIF) [file pcbi.1013523.s004.tif]

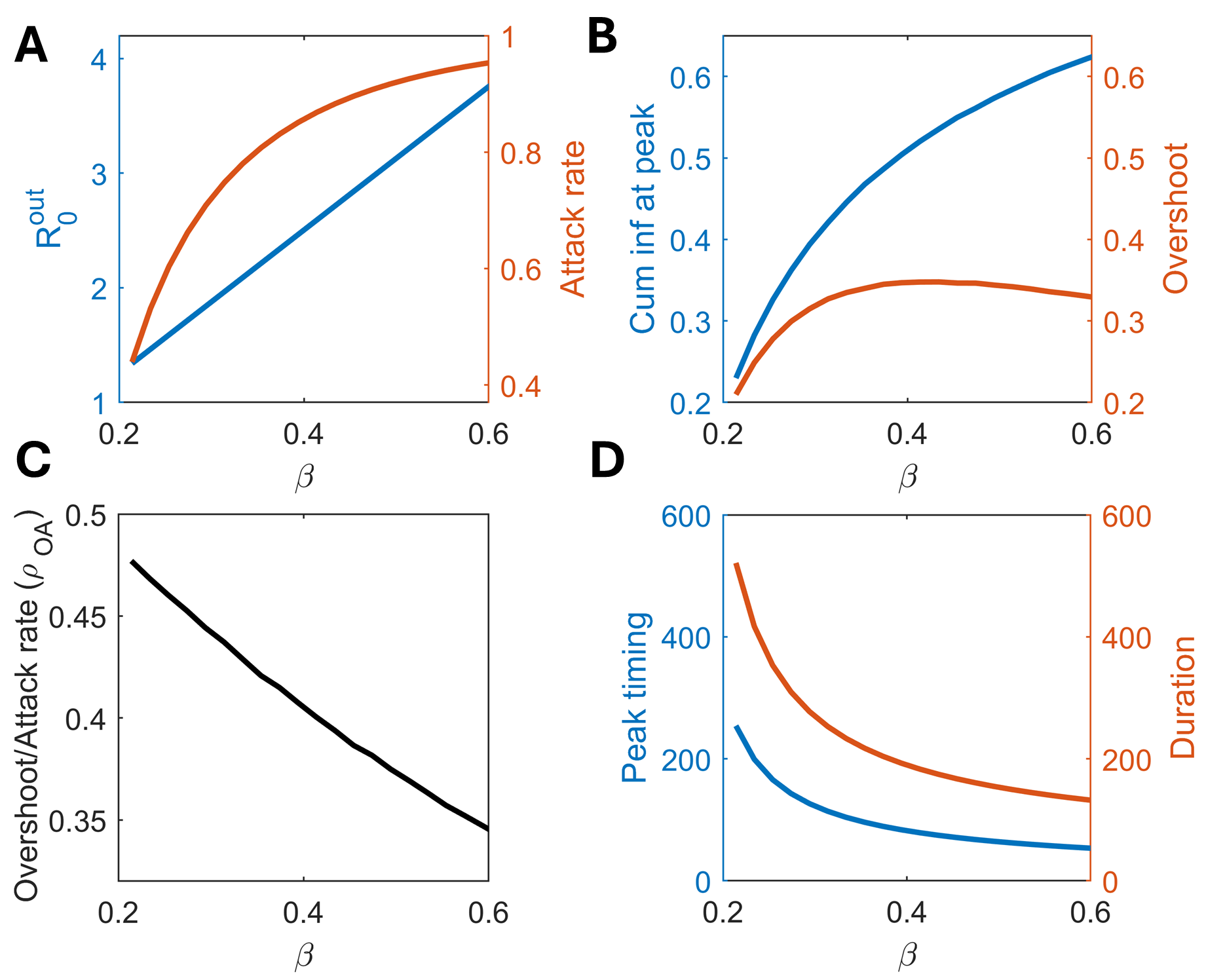

Supplement: S4 Fig — Effect of transmission rate via the free-living bacterial route (β) for the classical SIRB model on (A) ℛ0out and attack rate, (B) cumulative infections at peak (as a proportion of the total population) and epidemic overshoot, (C) the ratio of overshoot to attack rate (ρOA), and (D) peak timing and epidemic duration in the absence of bacteria-zooplankton association (σ=0). Intuitively, both ℛ0out and attack rate increase with β. While β increases cumulative infections at peak, there exists an upper bound on the overshoot. The ratio ρ OA decreases monotonically with increasing β due to the availability of fewer susceptible individuals for the overshoot (post-peak) phase. Both peak timing and epidemic duration consistently decrease as β increases. (TIF) [file pcbi.1013523.s005.tif]

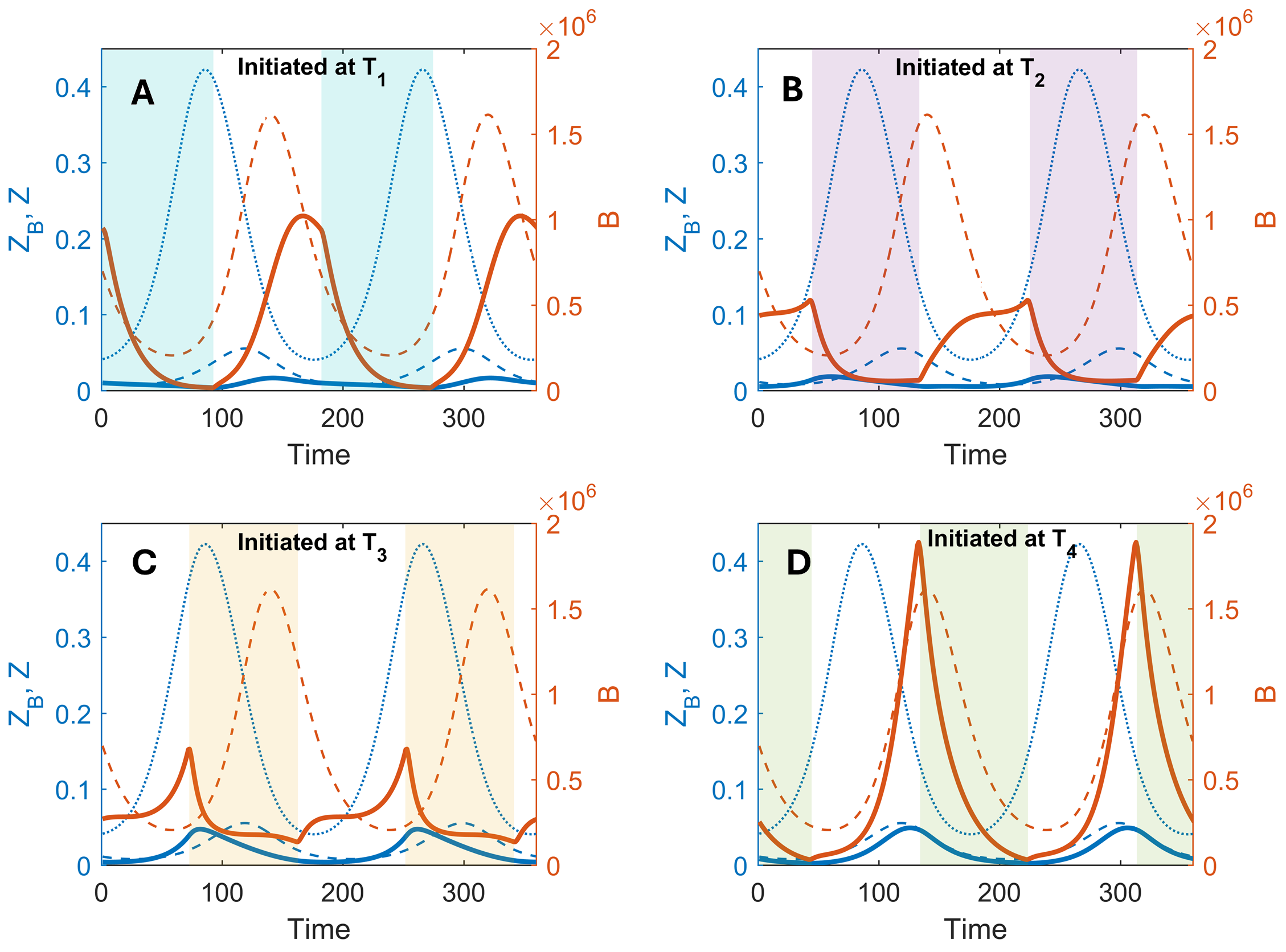

Supplement: S5 Fig — The formation of ZB (solid and dashed blue) depends on the density of B cells (solid and dashed red) in the water column during periods of Z abundance (dotted blue line) in both unfiltered (dashed) and filtered (solid) scenarios when filtration is initiated at T1-T4. The shaded regions indicate the filtration periods. Filtration reduces cholera infections in two ways: first, by restricting ZB formation through the reduction of B cell concentration during the periods Z abundance; and second, if ZB formation cannot be avoided, by preventing its ingestion. (TIF) [file pcbi.1013523.s006.tif]

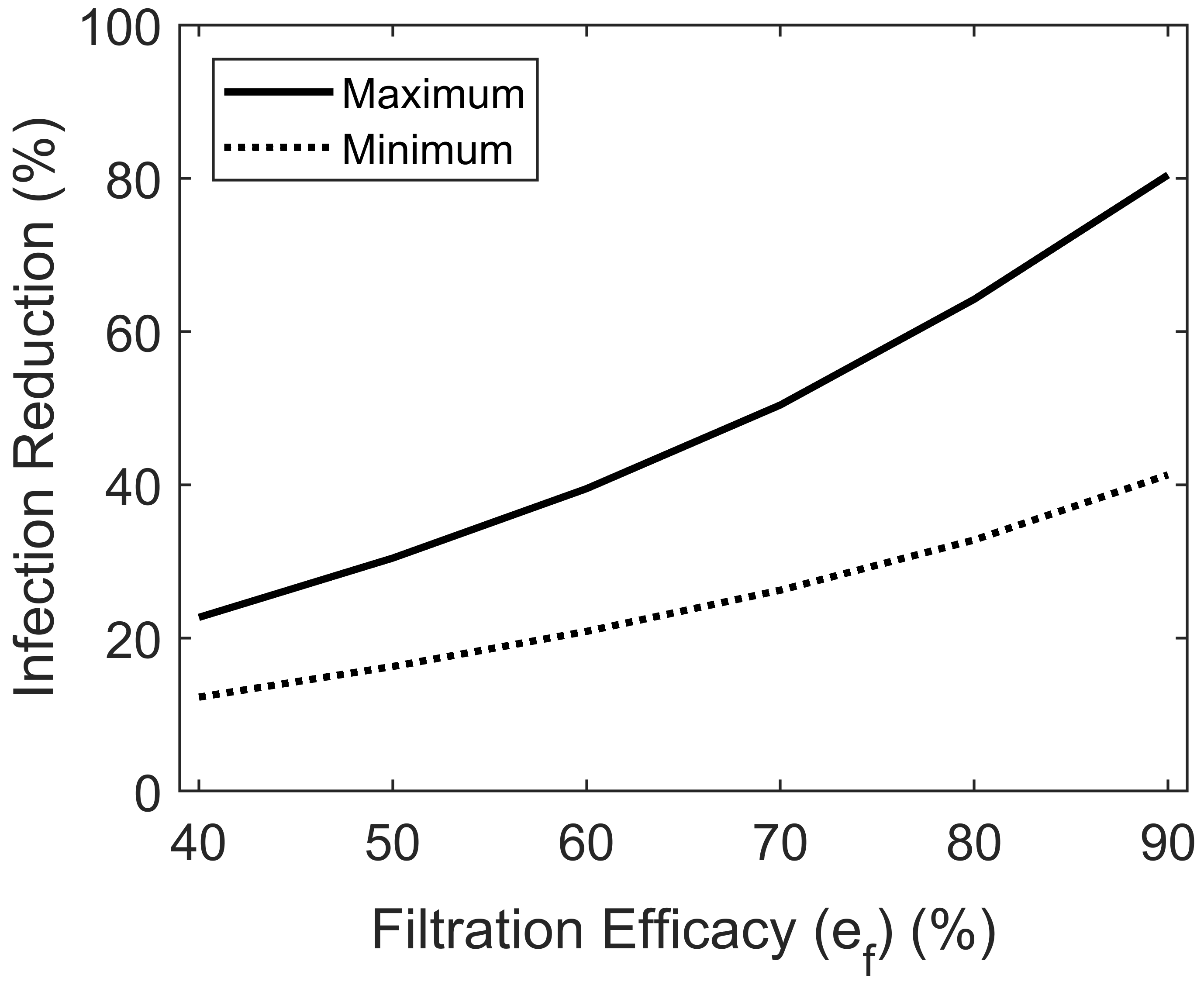

Supplement: S6 Fig — Cholera infection reduction (%) over a year under varying filtration efficacy (ef). The solid line denotes the maximum reduction and the dotted line denotes the minimum, calculated across all possible filtration initiation timings. (TIF) [file pcbi.1013523.s007.tif]
